# Supplementary figures and images for: Identification of epigenetic modulators as determinants of nuclear size and shape
Source: eLife. 2023 May 23;12:e80653. doi: 10.7554/eLife.80653 (PMC10259489; doi:10.7554/eLife.80653)

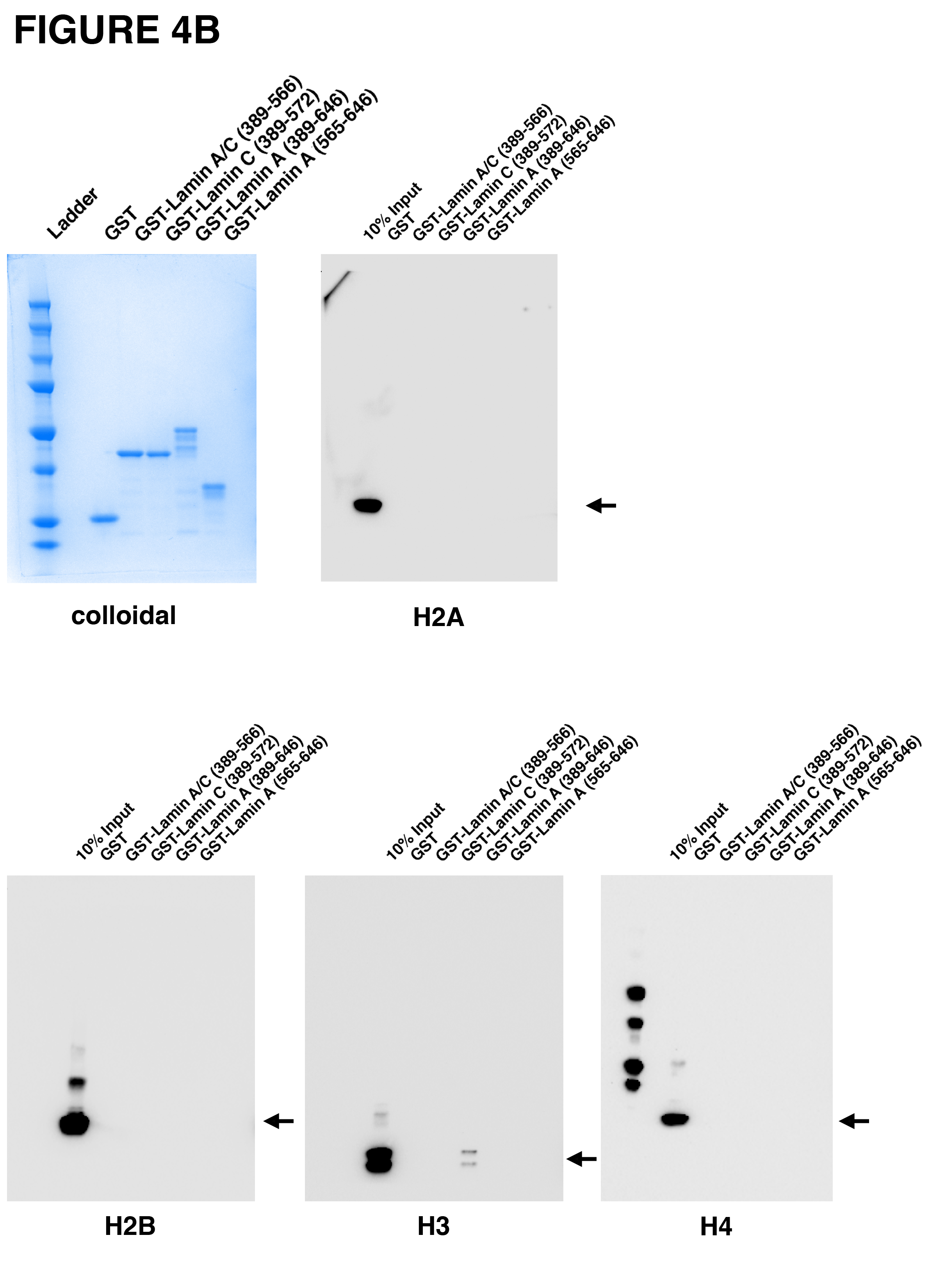

Supplement: Figure 4—source data 1. [file elife-80653-fig4-data1.zip › Figure 4-source data 1 /Figure 4B.tif]

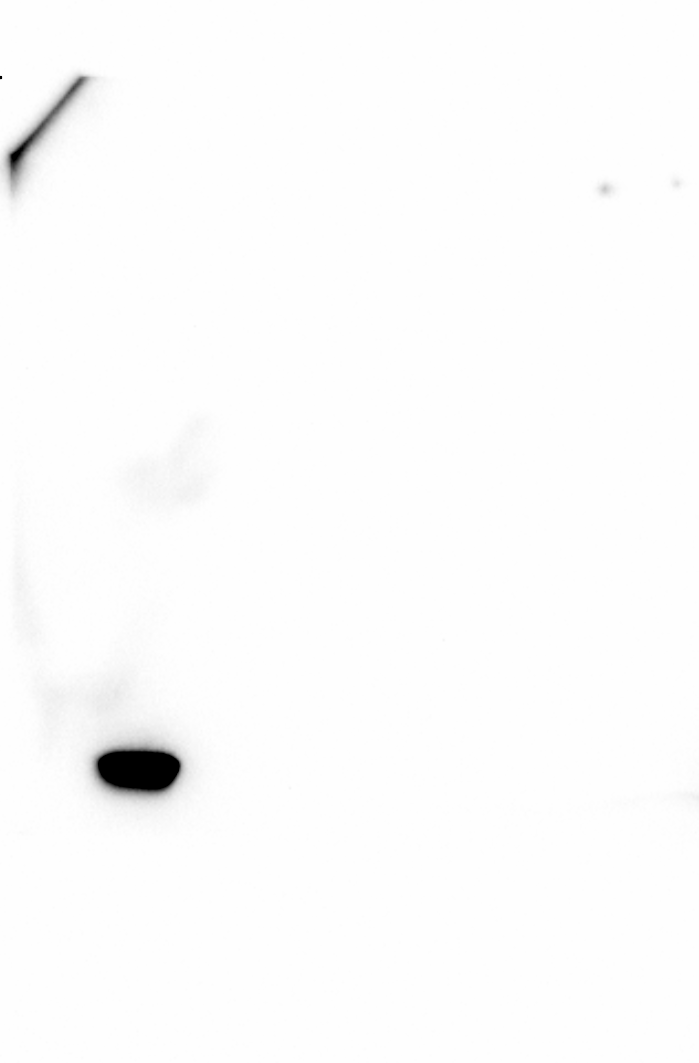

Supplement: Figure 4—source data 1. [file elife-80653-fig4-data1.zip › Figure 4-source data 1 /ORIGINAL FILES/FIgure 4B H2A.tif]

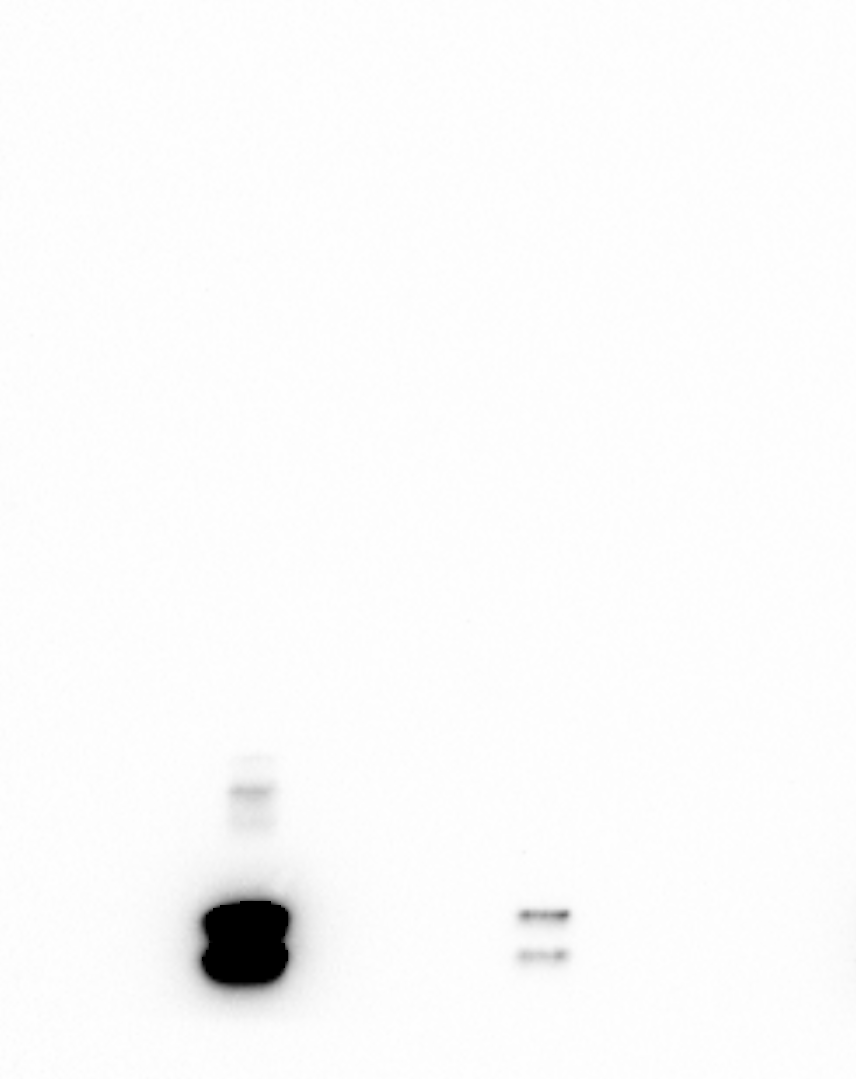

Supplement: Figure 4—source data 1. [file elife-80653-fig4-data1.zip › Figure 4-source data 1 /ORIGINAL FILES/Figure 4B H3.tif]

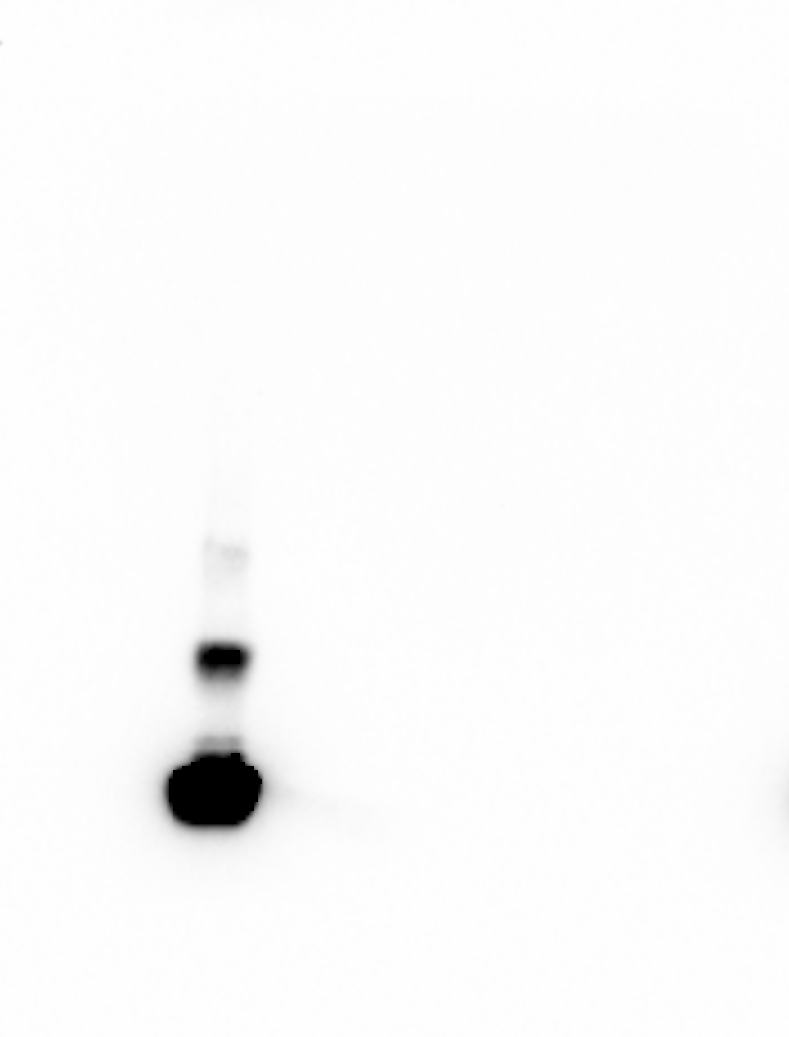

Supplement: Figure 4—source data 1. [file elife-80653-fig4-data1.zip › Figure 4-source data 1 /ORIGINAL FILES/FIgure 4B H2B.tif]

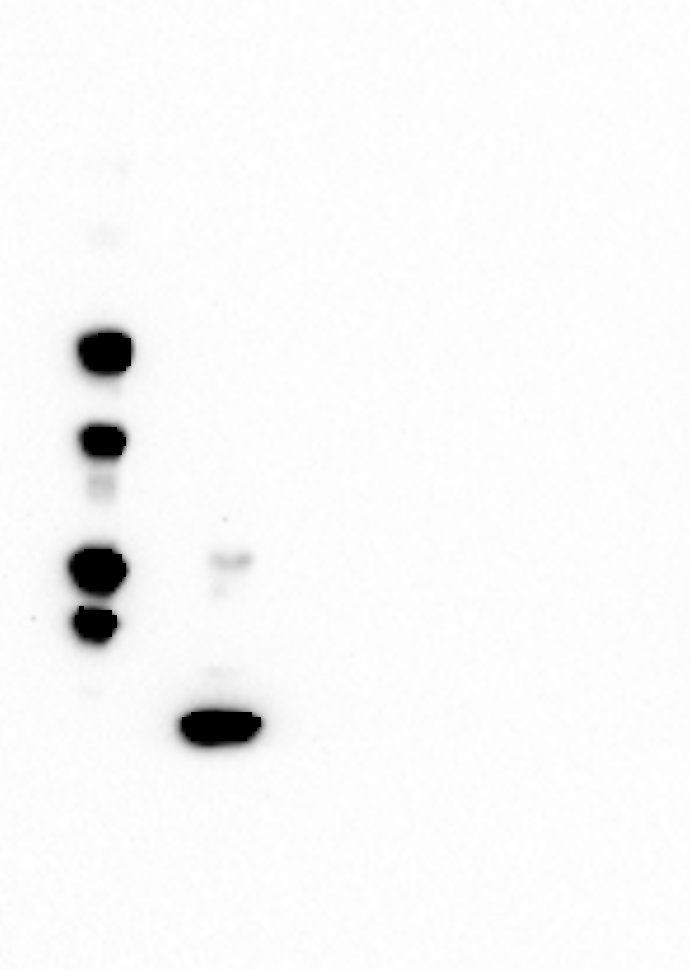

Supplement: Figure 4—source data 1. [file elife-80653-fig4-data1.zip › Figure 4-source data 1 /ORIGINAL FILES/Figure 4B H4.tif]

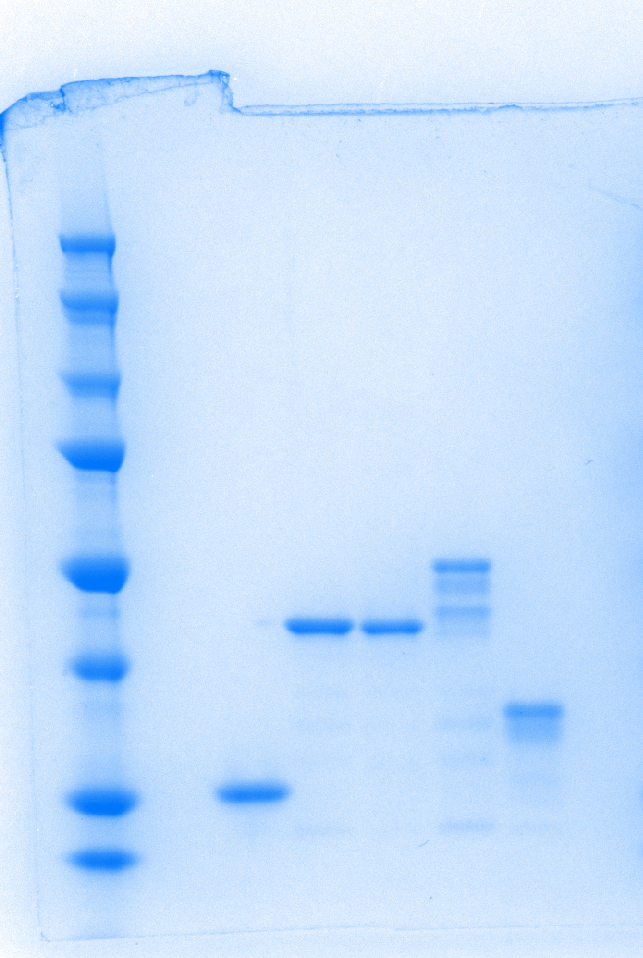

Supplement: Figure 4—source data 1. [file elife-80653-fig4-data1.zip › Figure 4-source data 1 /ORIGINAL FILES/Figure 4B colloidal.tif]

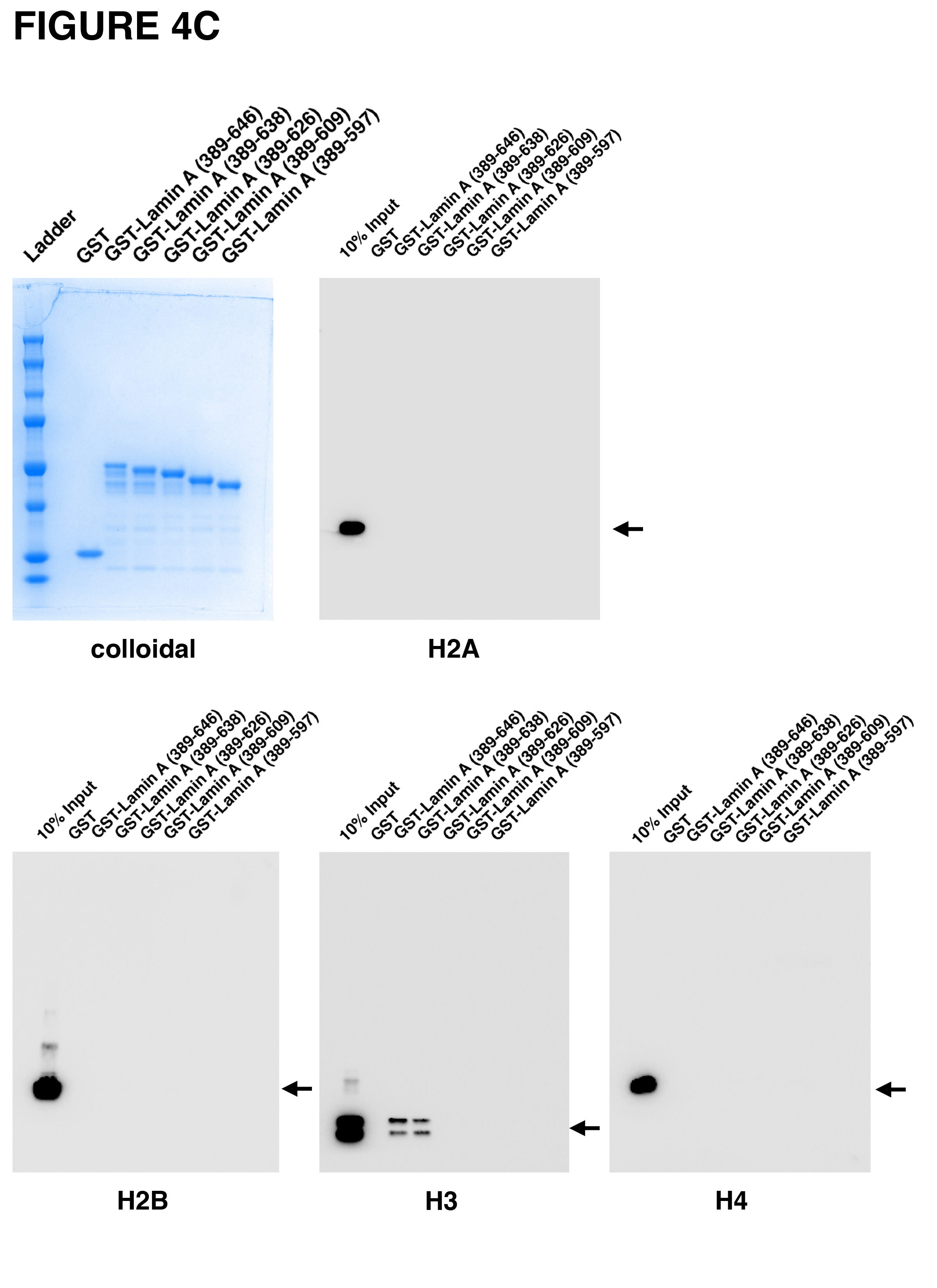

Supplement: Figure 4—source data 2. [file elife-80653-fig4-data2.zip › Figure 4-source data 2/Figure 4C.tif]

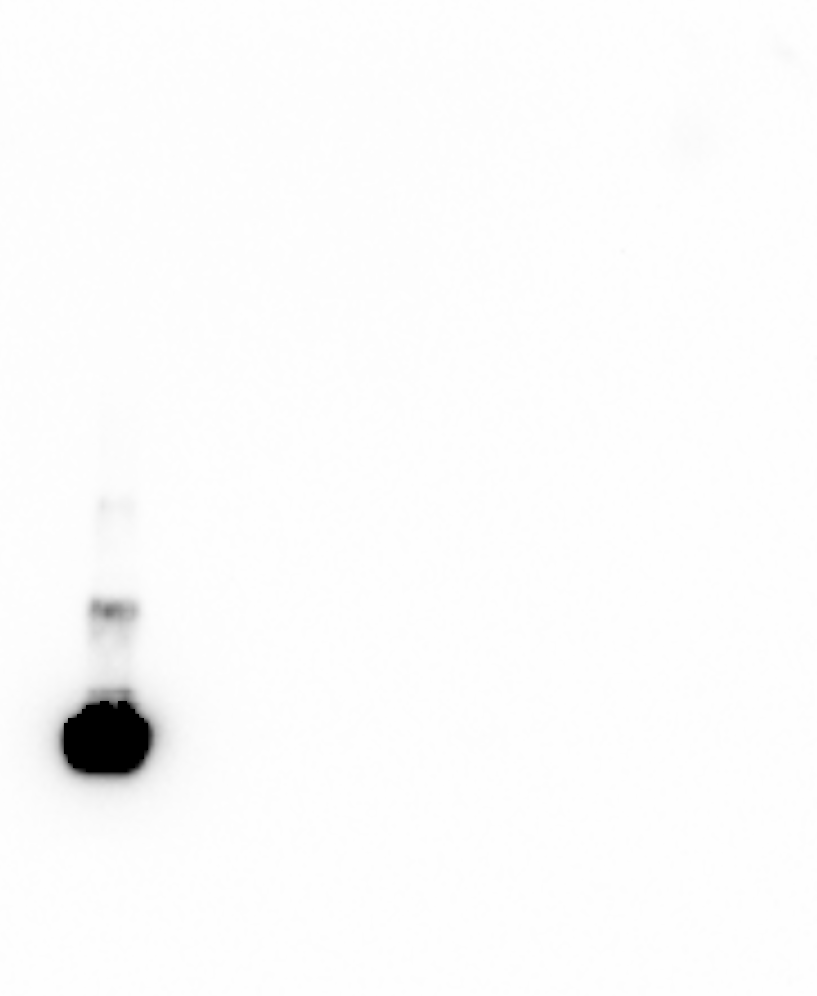

Supplement: Figure 4—source data 2. [file elife-80653-fig4-data2.zip › Figure 4-source data 2/ORIGINAL FILES/Figure 4C H2B.tif]

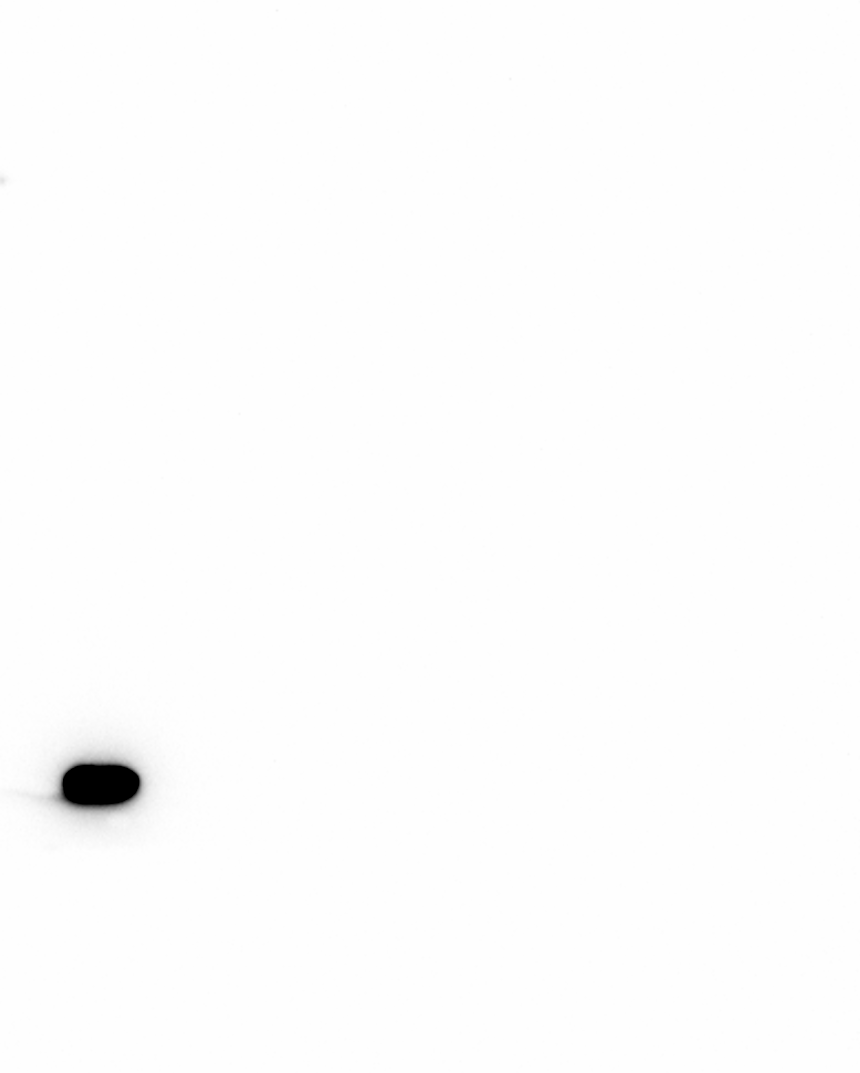

Supplement: Figure 4—source data 2. [file elife-80653-fig4-data2.zip › Figure 4-source data 2/ORIGINAL FILES/Figure 4C H2A.tif]

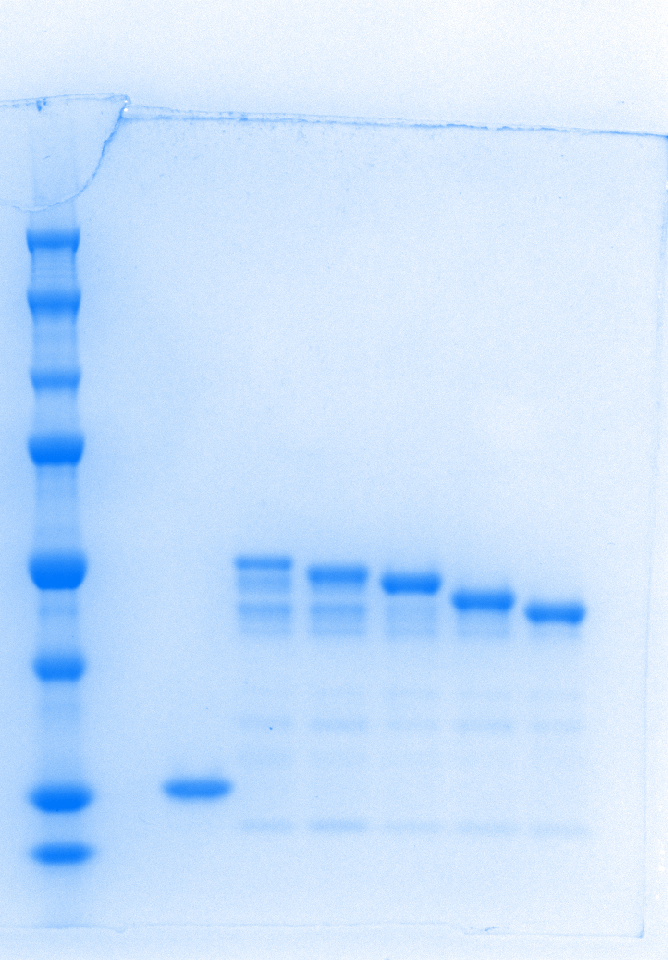

Supplement: Figure 4—source data 2. [file elife-80653-fig4-data2.zip › Figure 4-source data 2/ORIGINAL FILES/FIgure 4C colloidal.tif]

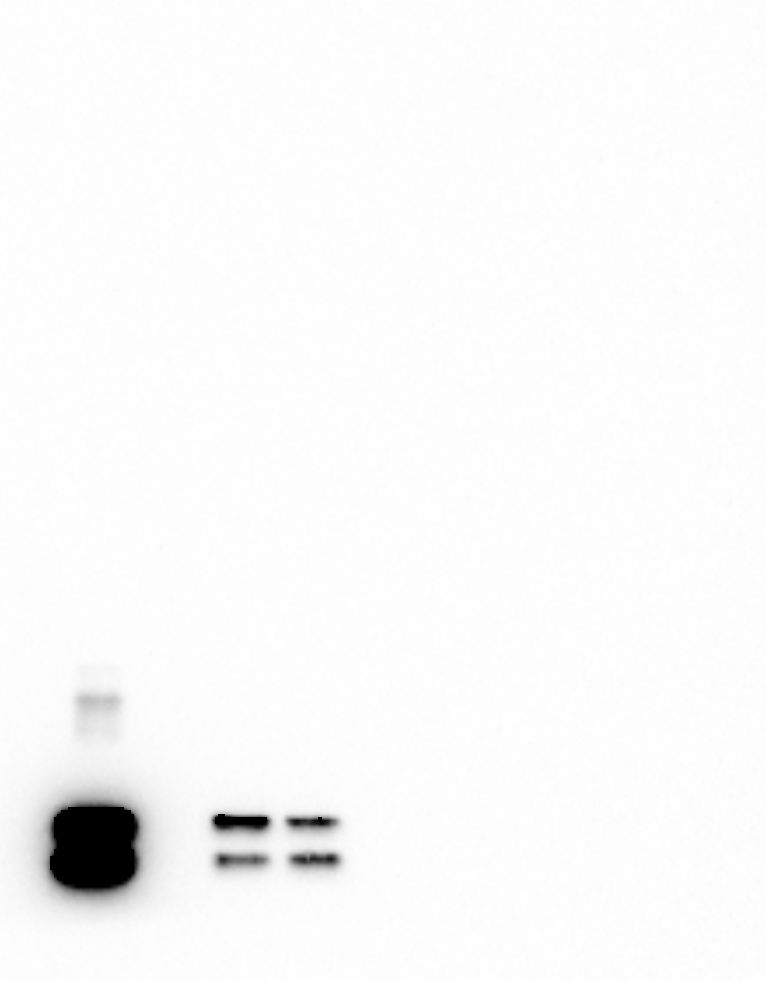

Supplement: Figure 4—source data 2. [file elife-80653-fig4-data2.zip › Figure 4-source data 2/ORIGINAL FILES/Figure 4C H3.tif]

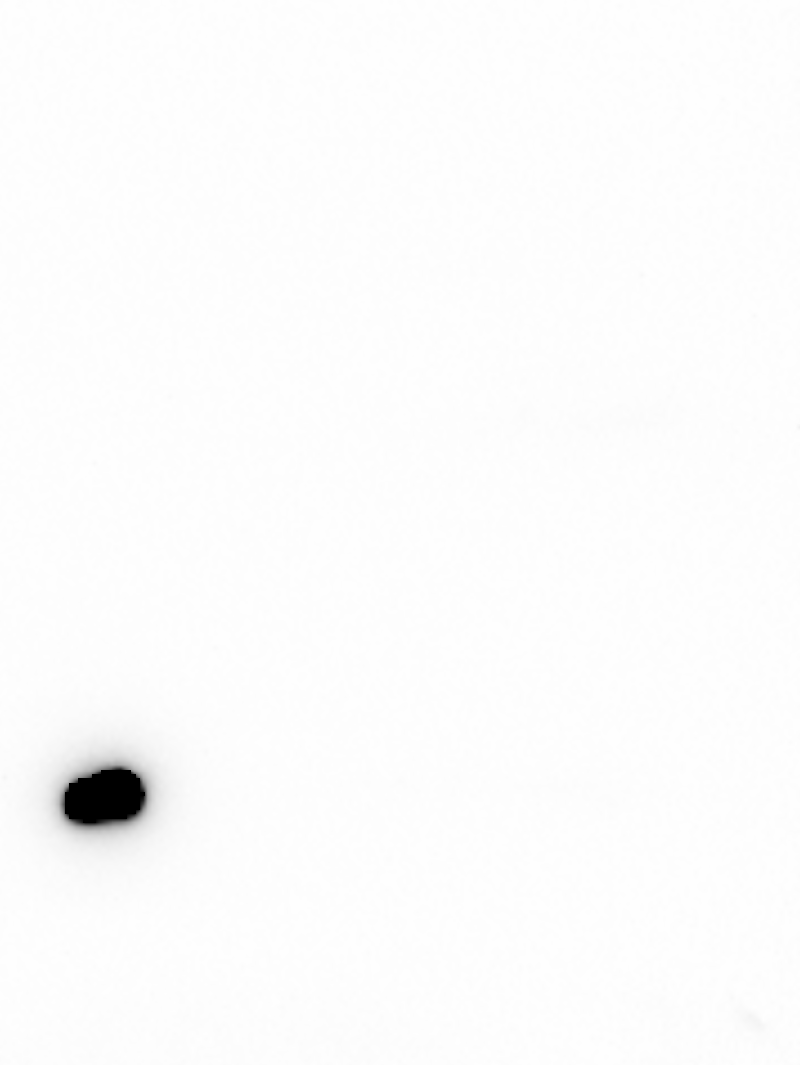

Supplement: Figure 4—source data 2. [file elife-80653-fig4-data2.zip › Figure 4-source data 2/ORIGINAL FILES/Figure 4C H4.tif]

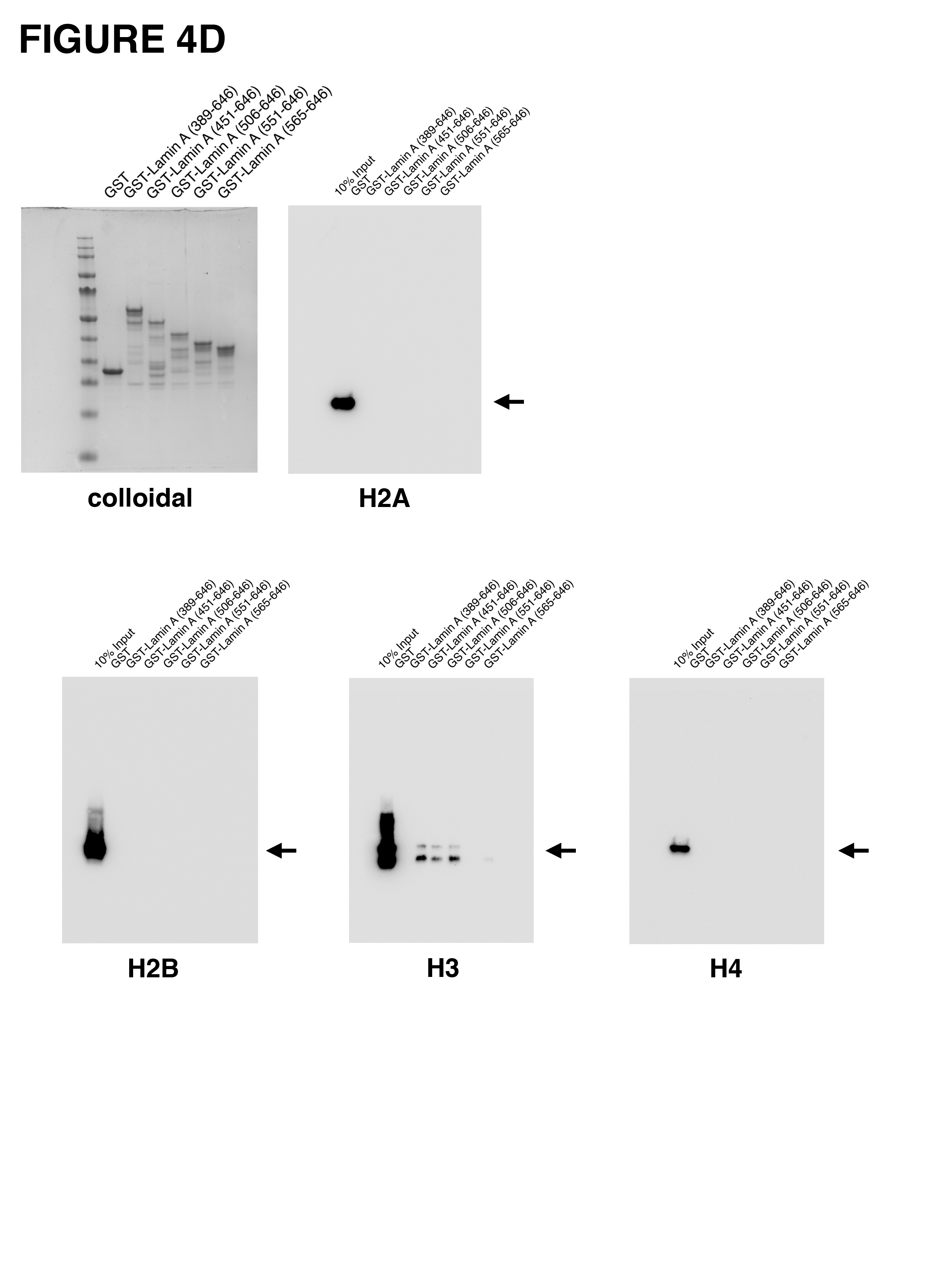

Supplement: Figure 4—source data 3. [file elife-80653-fig4-data3.zip › Figure 4-source data 3/Figure 4D.tif]

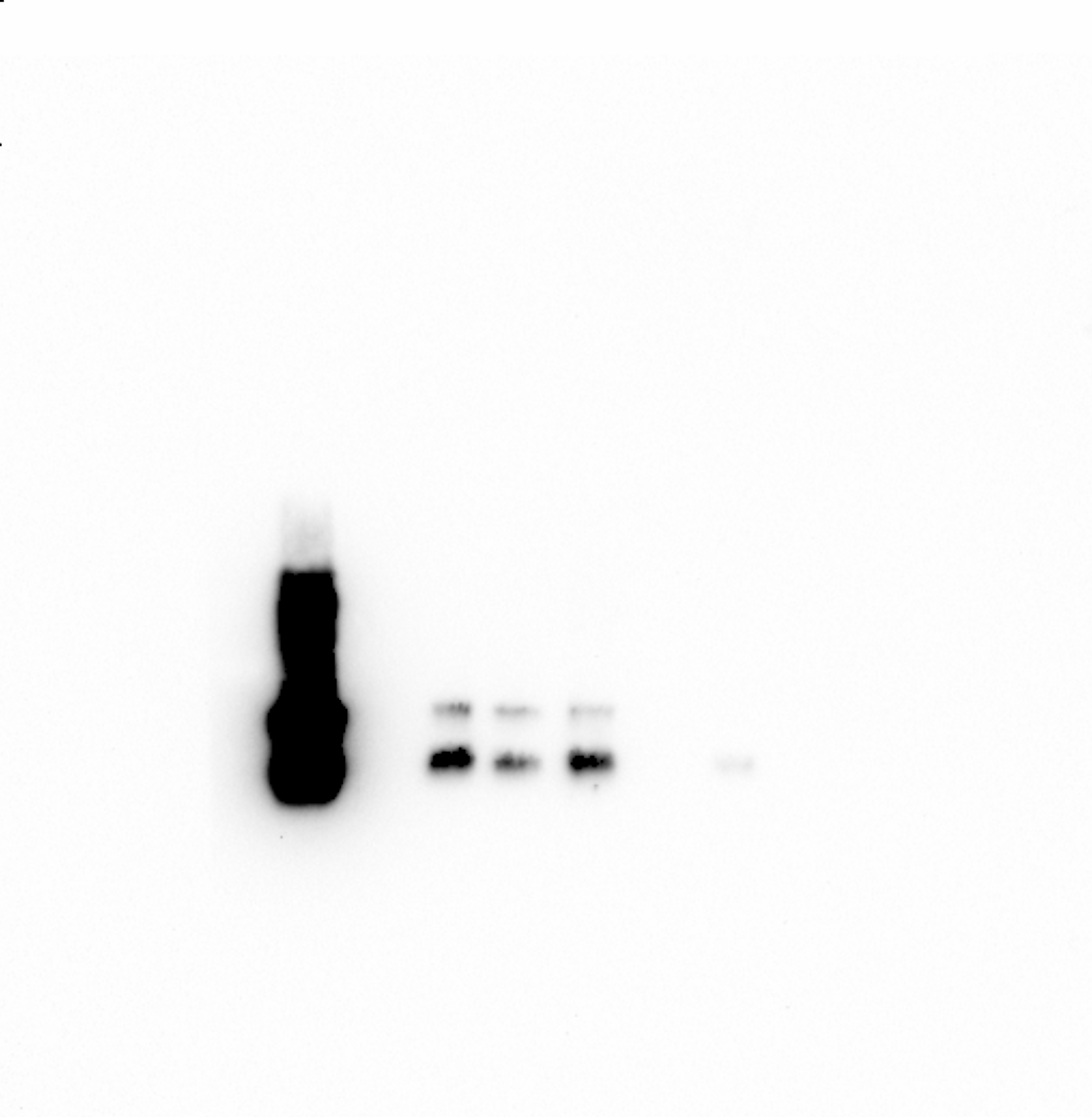

Supplement: Figure 4—source data 3. [file elife-80653-fig4-data3.zip › Figure 4-source data 3/ORIGINAL FILES /Figure 4D H3.tif]

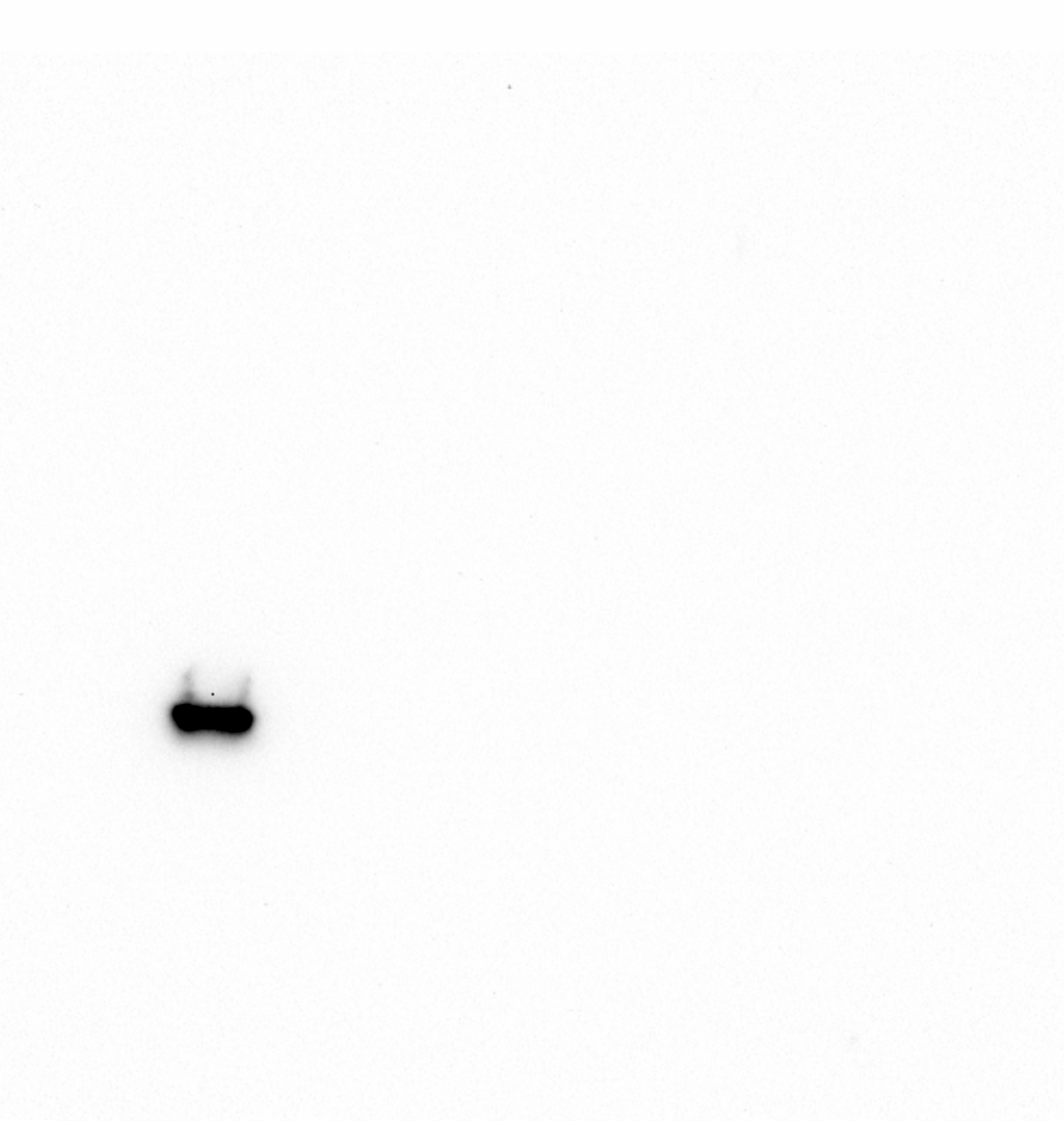

Supplement: Figure 4—source data 3. [file elife-80653-fig4-data3.zip › Figure 4-source data 3/ORIGINAL FILES /Figure 4D H4.tif]

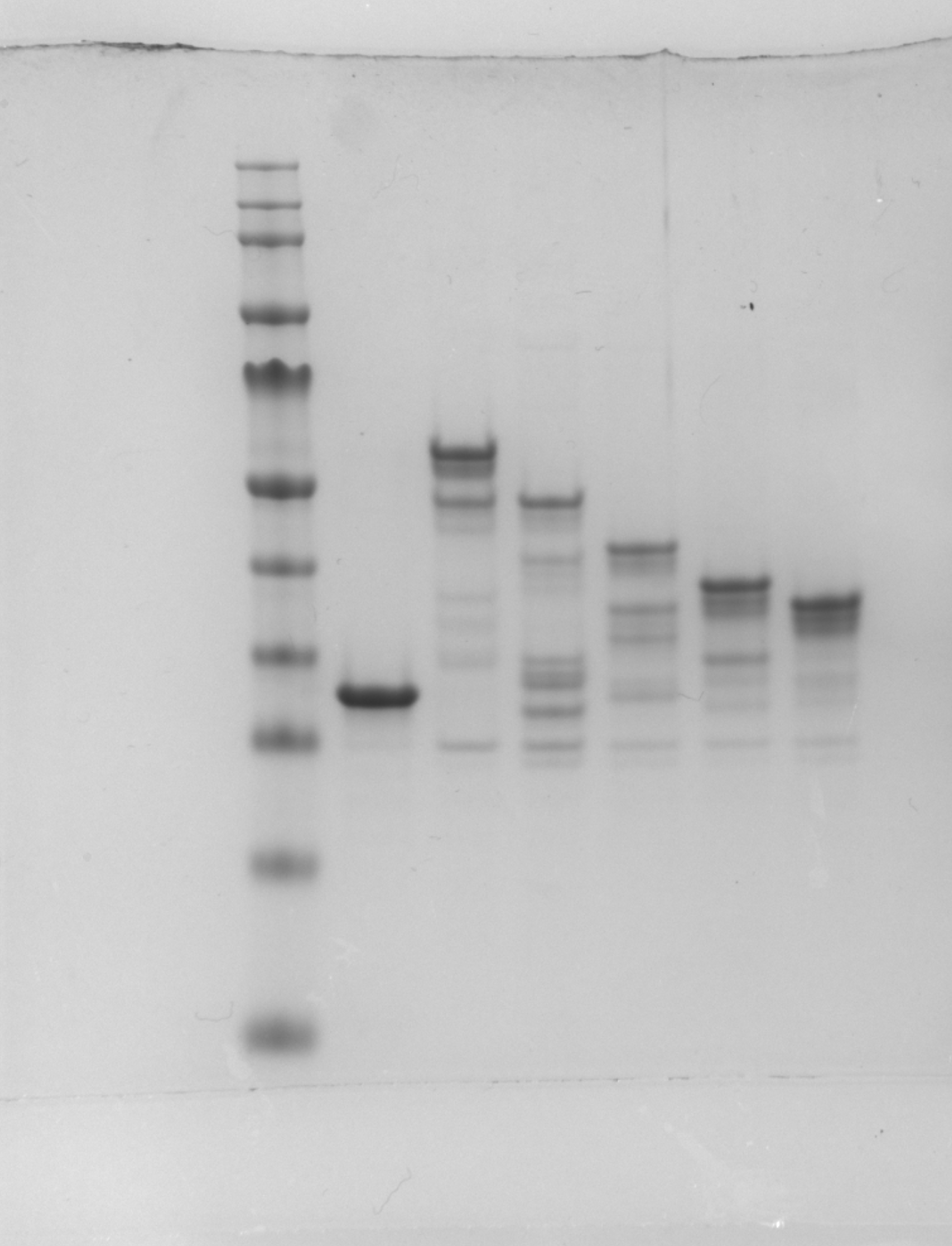

Supplement: Figure 4—source data 3. [file elife-80653-fig4-data3.zip › Figure 4-source data 3/ORIGINAL FILES /Figure 4D colloidal.tif]

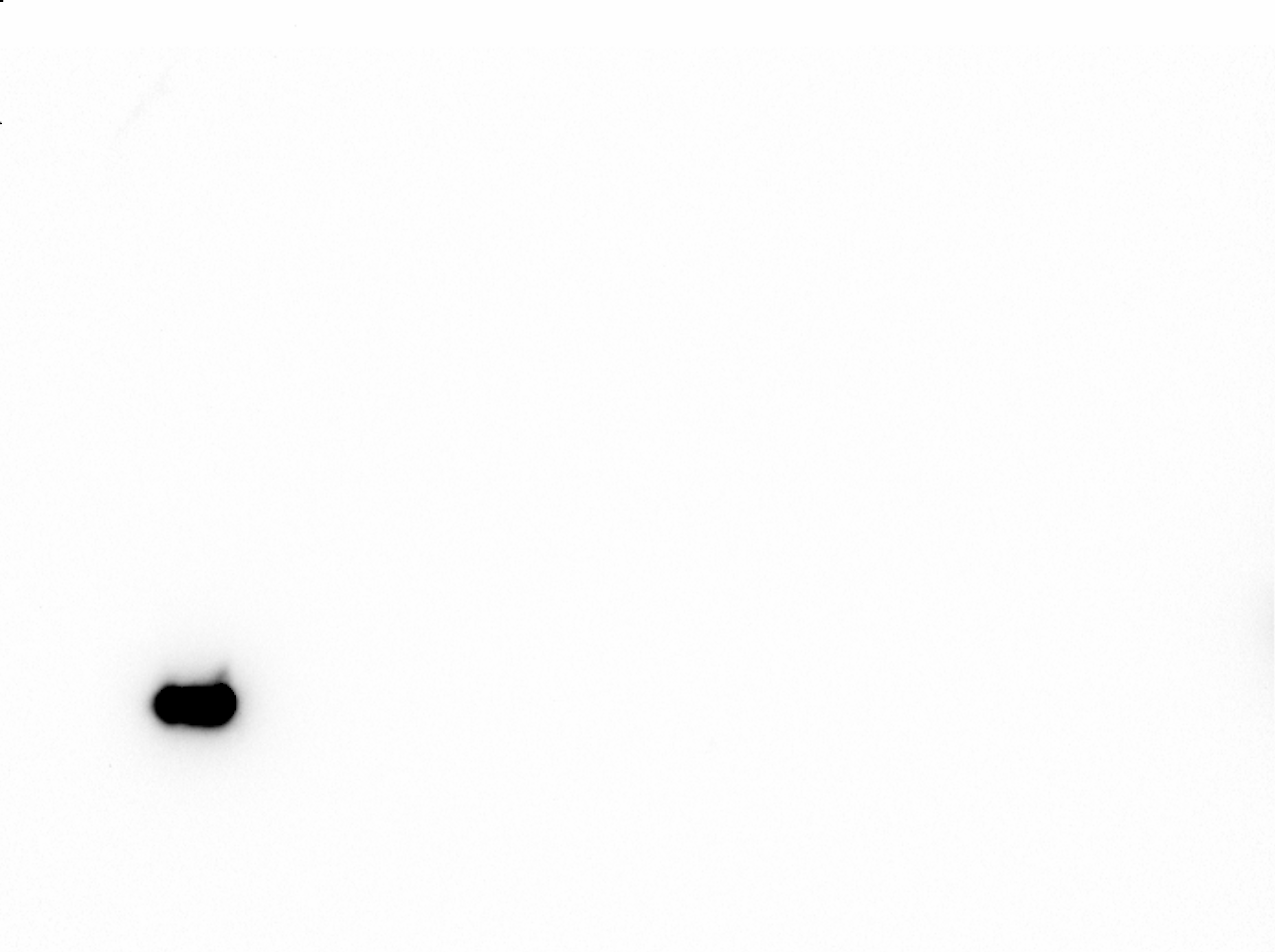

Supplement: Figure 4—source data 3. [file elife-80653-fig4-data3.zip › Figure 4-source data 3/ORIGINAL FILES /Figure 4D H2A.tif]

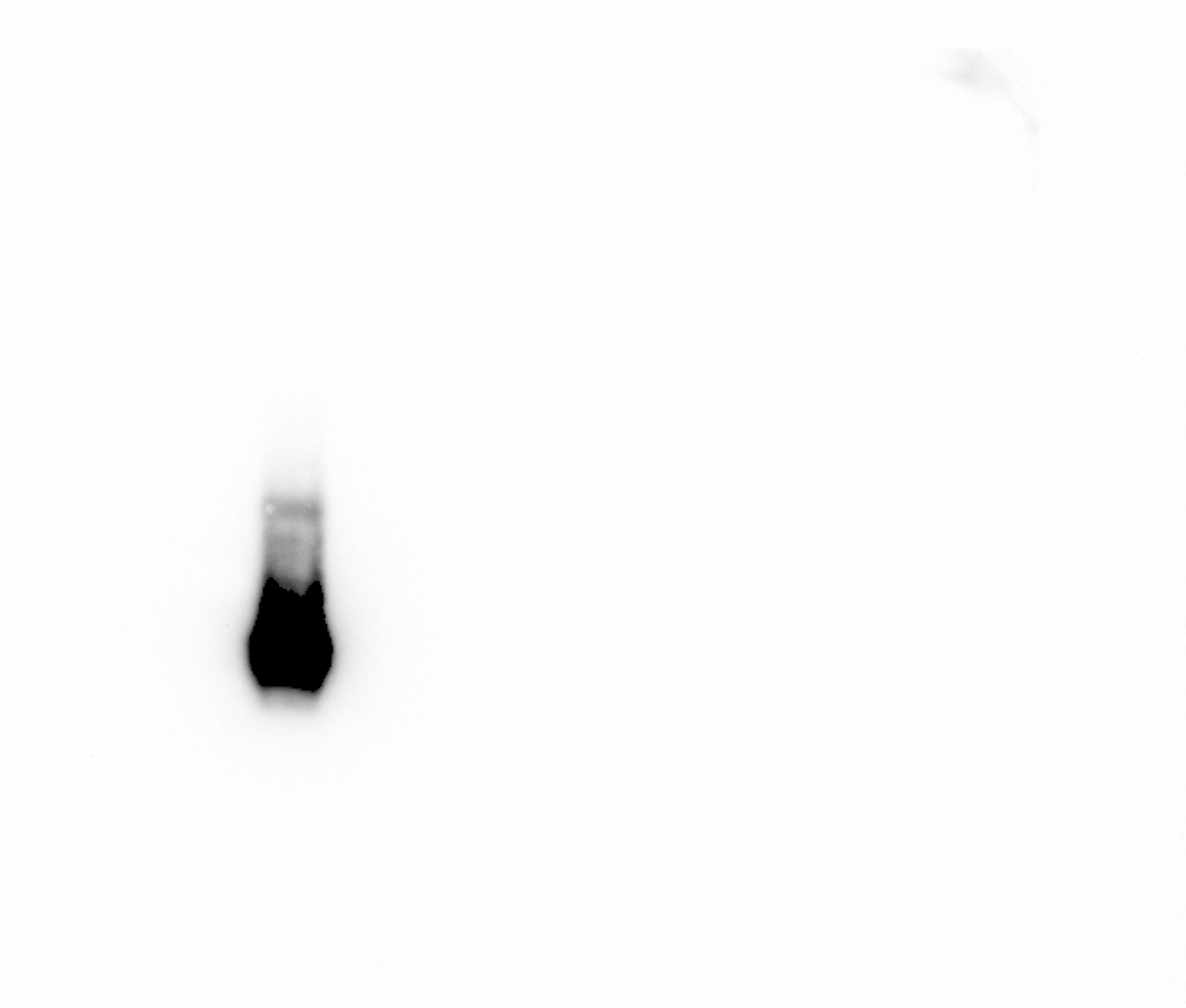

Supplement: Figure 4—source data 3. [file elife-80653-fig4-data3.zip › Figure 4-source data 3/ORIGINAL FILES /Figure 4D H2B.tif]

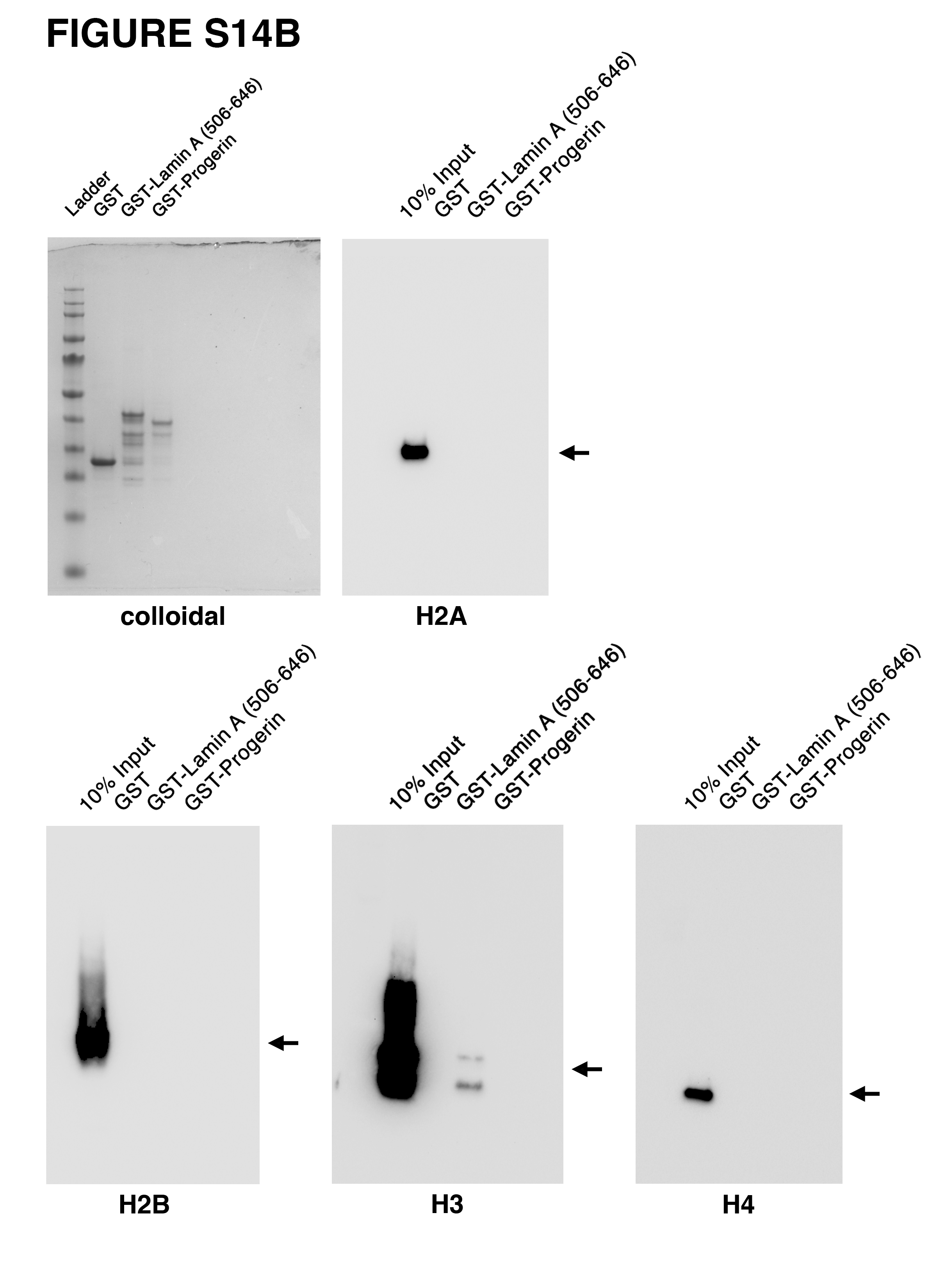

Supplement: Figure 4—figure supplement 1—source data 1. [file elife-80653-fig4-figsupp1-data1.zip › Figure 4 - figure supplement 1 -source data 1 /Paenl B.tif]

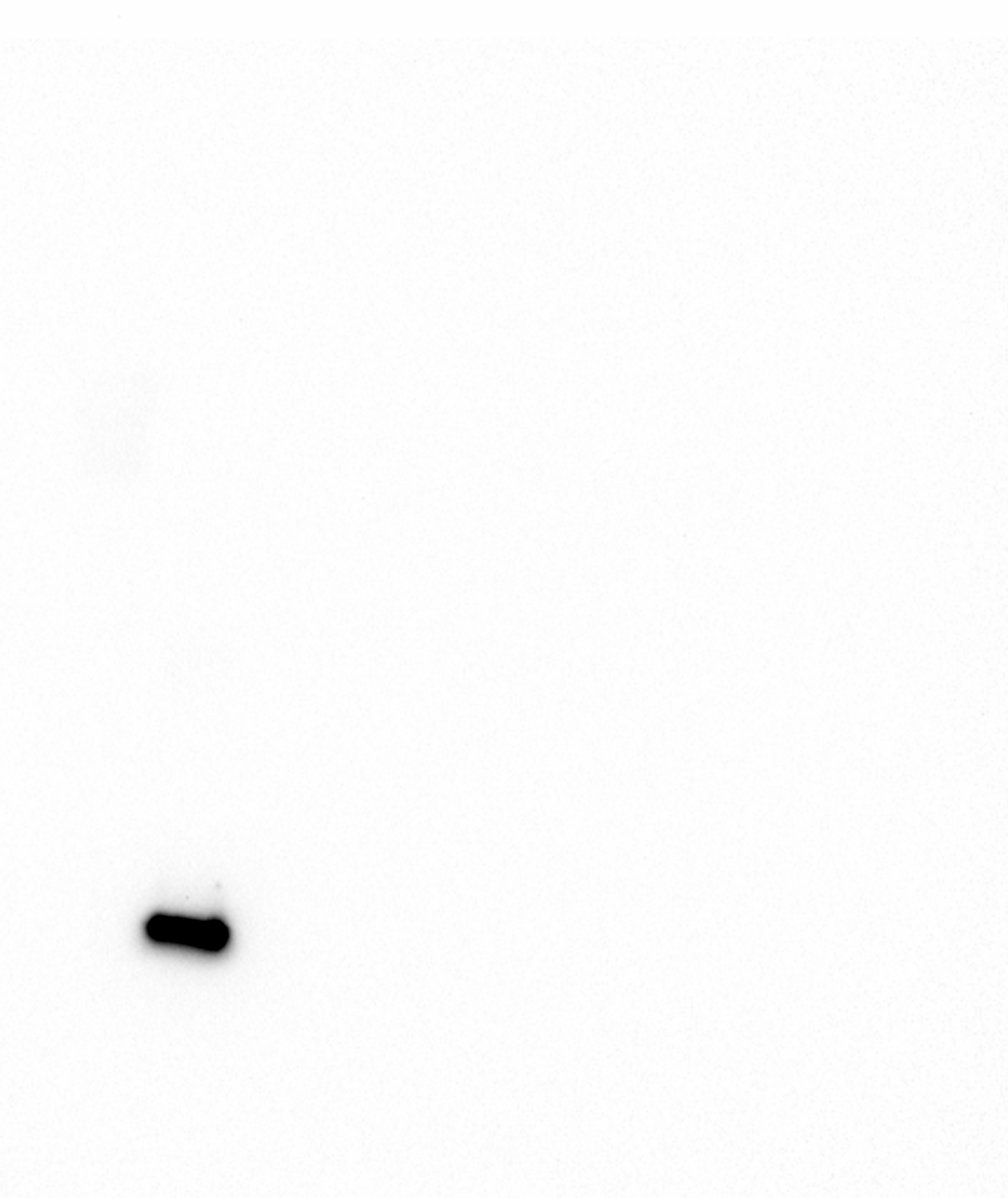

Supplement: Figure 4—figure supplement 1—source data 1. [file elife-80653-fig4-figsupp1-data1.zip › Figure 4 - figure supplement 1 -source data 1 /ORIGINAL FILES/Paenl B H4.tif]

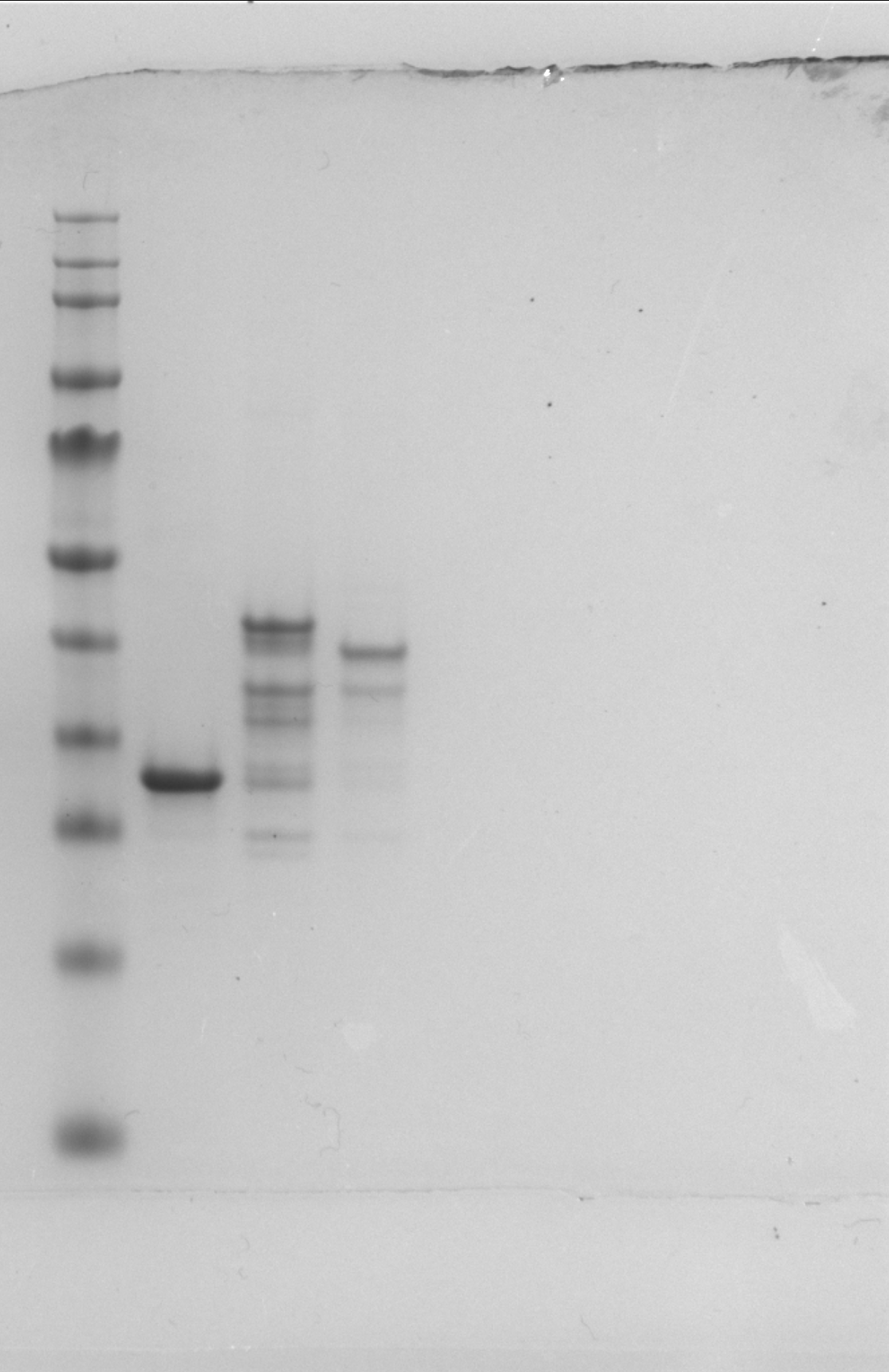

Supplement: Figure 4—figure supplement 1—source data 1. [file elife-80653-fig4-figsupp1-data1.zip › Figure 4 - figure supplement 1 -source data 1 /ORIGINAL FILES/Panel B colloidal.tif]

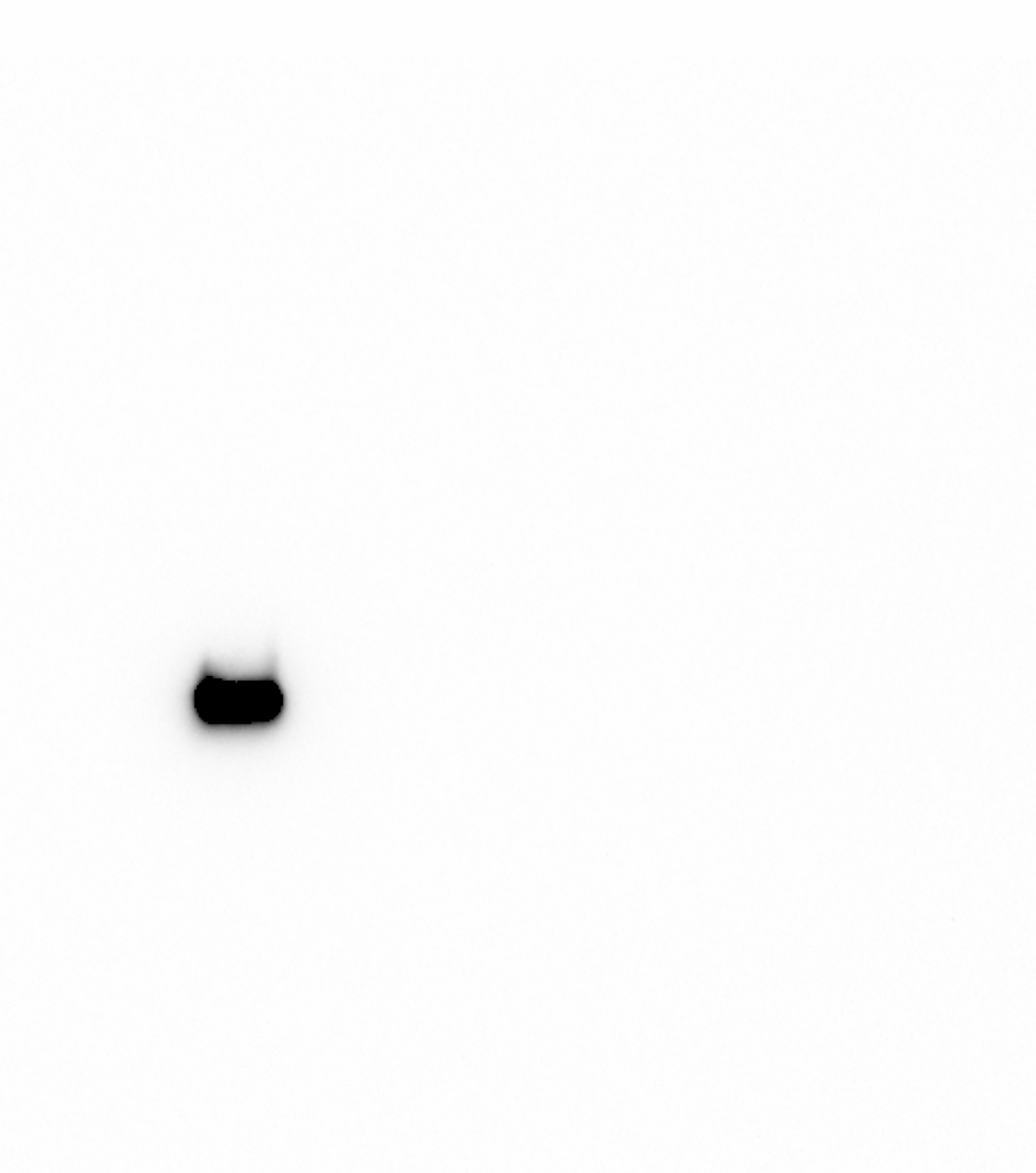

Supplement: Figure 4—figure supplement 1—source data 1. [file elife-80653-fig4-figsupp1-data1.zip › Figure 4 - figure supplement 1 -source data 1 /ORIGINAL FILES/Panel B H2A.tif]

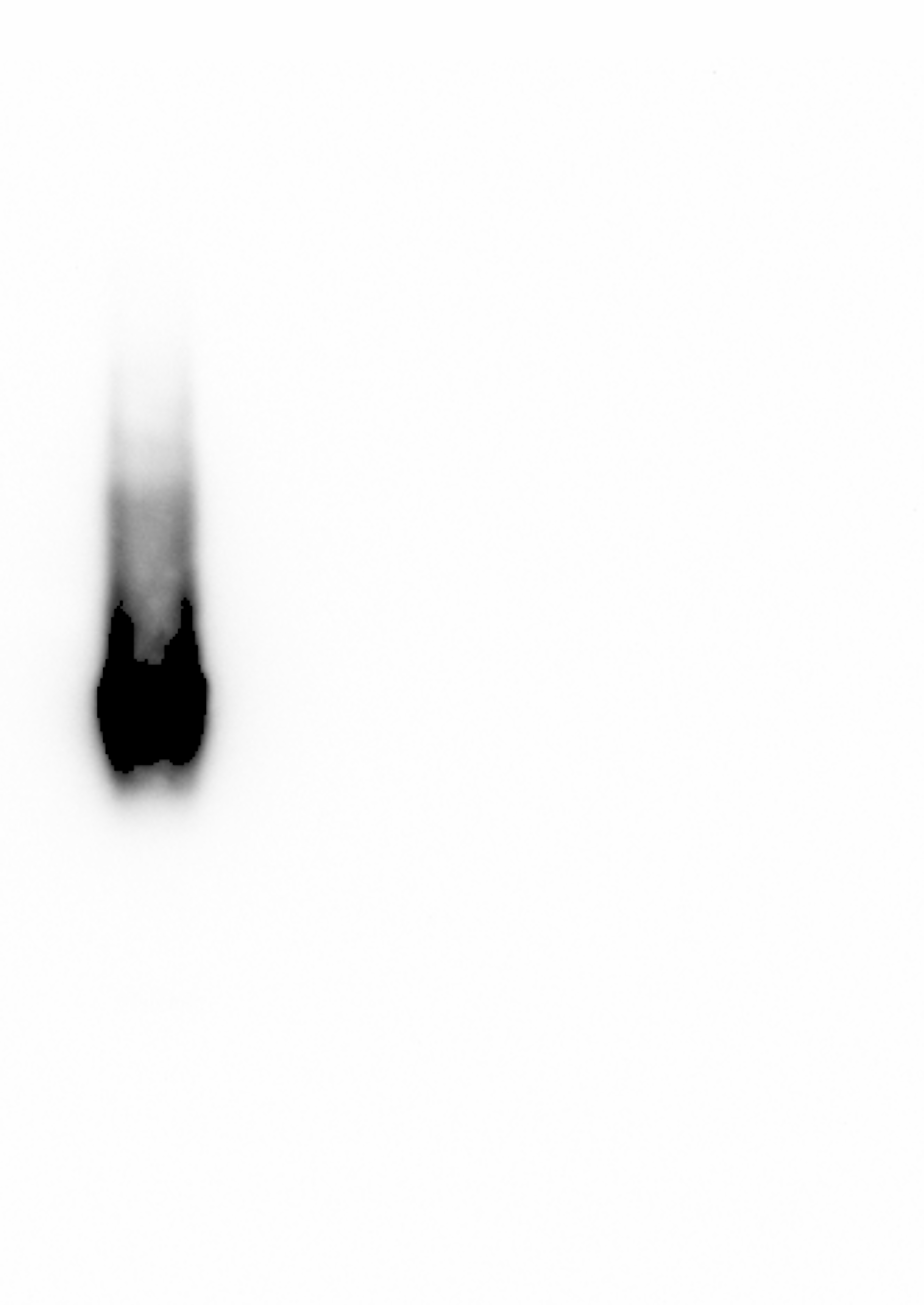

Supplement: Figure 4—figure supplement 1—source data 1. [file elife-80653-fig4-figsupp1-data1.zip › Figure 4 - figure supplement 1 -source data 1 /ORIGINAL FILES/Panel B H2B.tif]

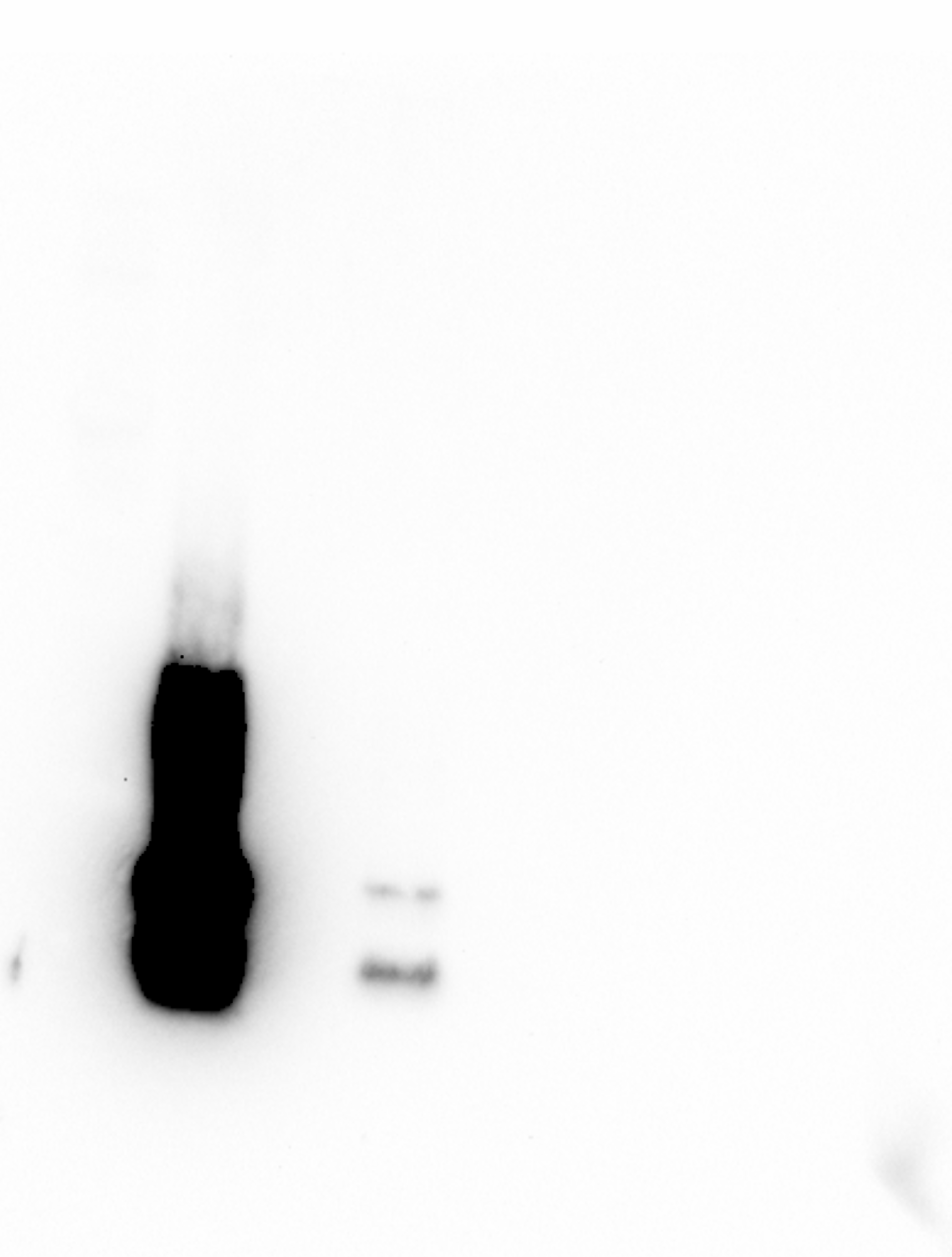

Supplement: Figure 4—figure supplement 1—source data 1. [file elife-80653-fig4-figsupp1-data1.zip › Figure 4 - figure supplement 1 -source data 1 /ORIGINAL FILES/Panel B H3.tif]

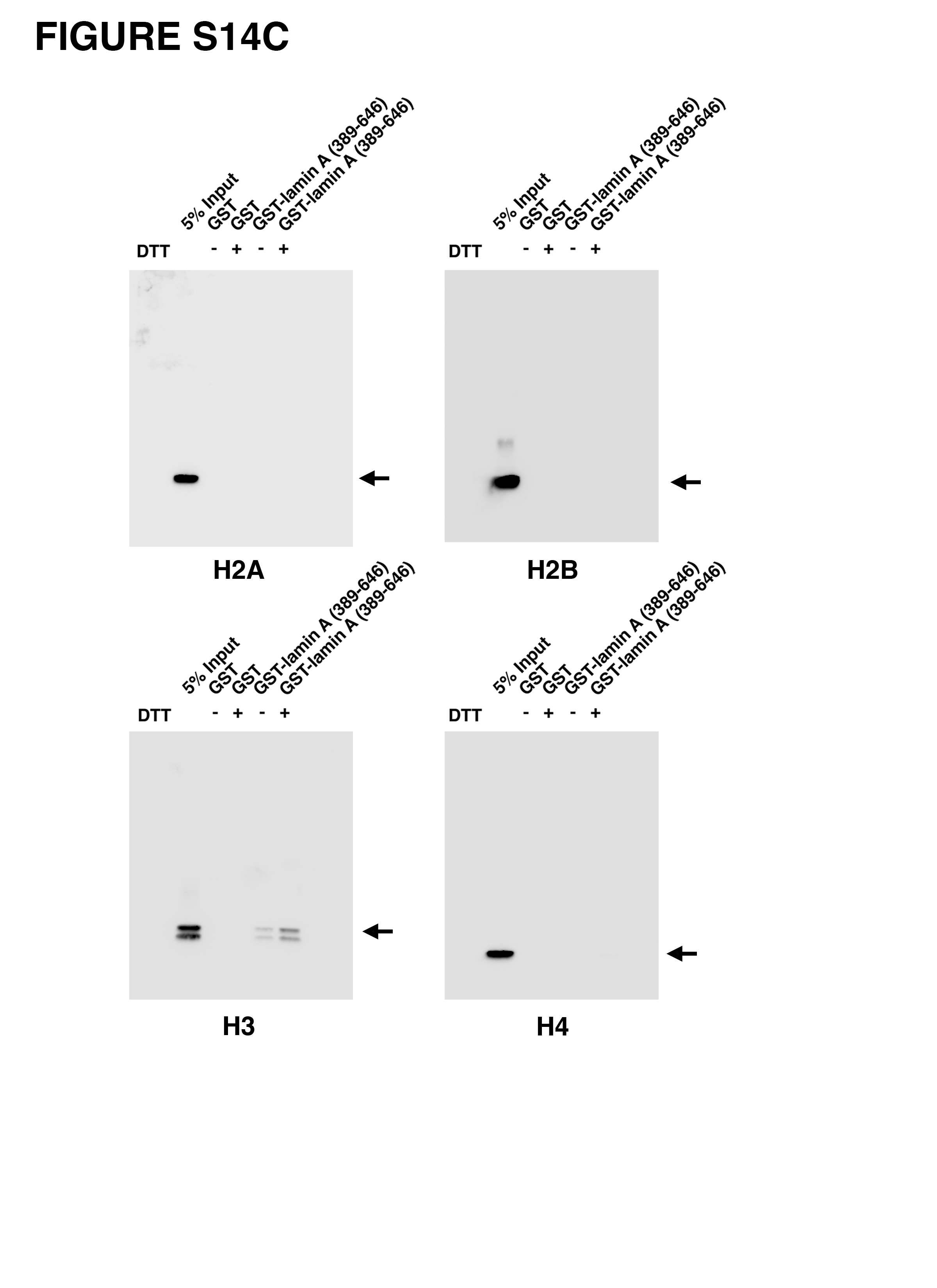

Supplement: Figure 4—figure supplement 1—source data 2. [file elife-80653-fig4-figsupp1-data2.zip › Figure 4-figure supplement 2 -source data 1/Panel C.tif]

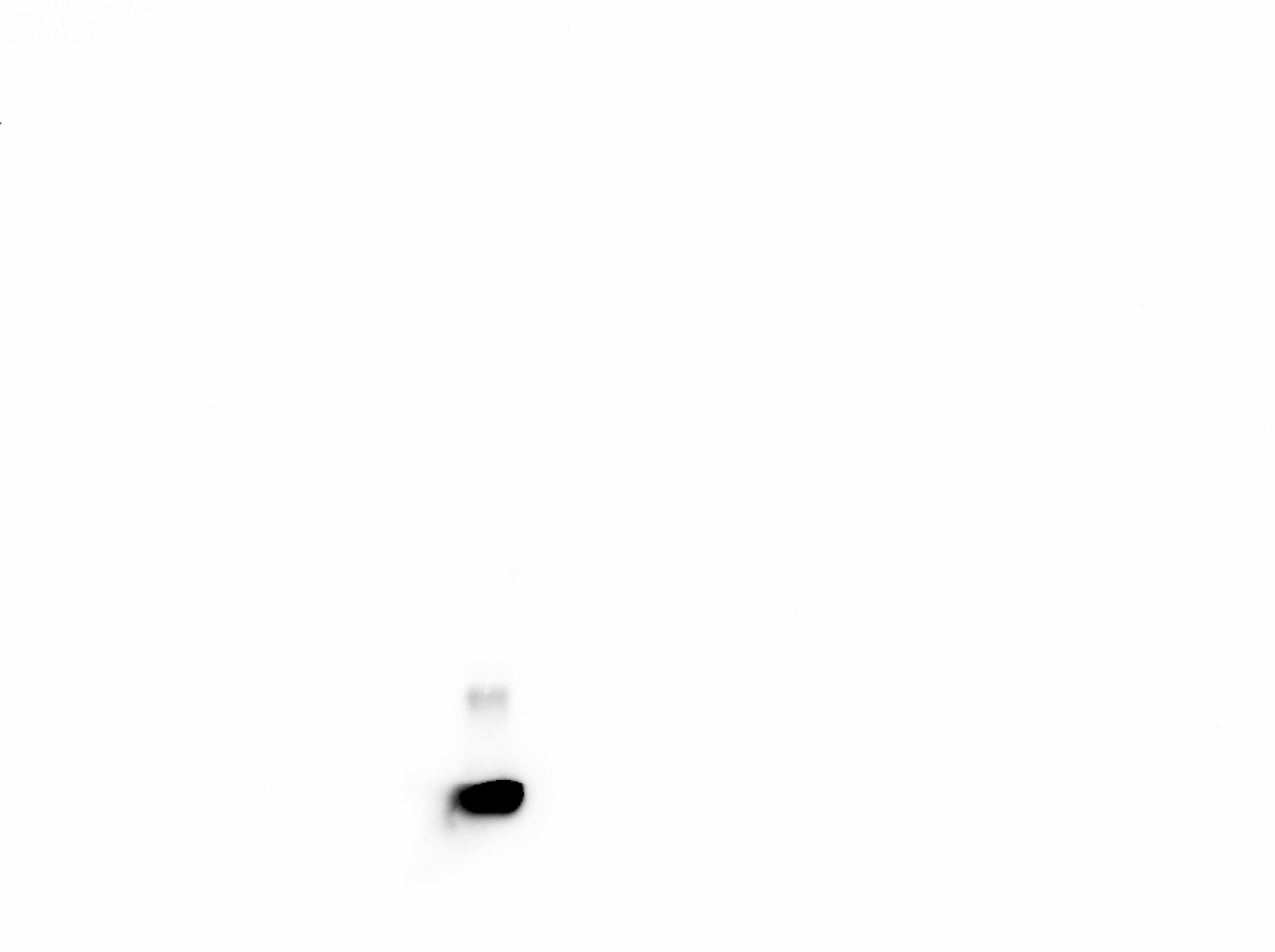

Supplement: Figure 4—figure supplement 1—source data 2. [file elife-80653-fig4-figsupp1-data2.zip › Figure 4-figure supplement 2 -source data 1/ORIGINAL FILES/Panel C H2B.tif]

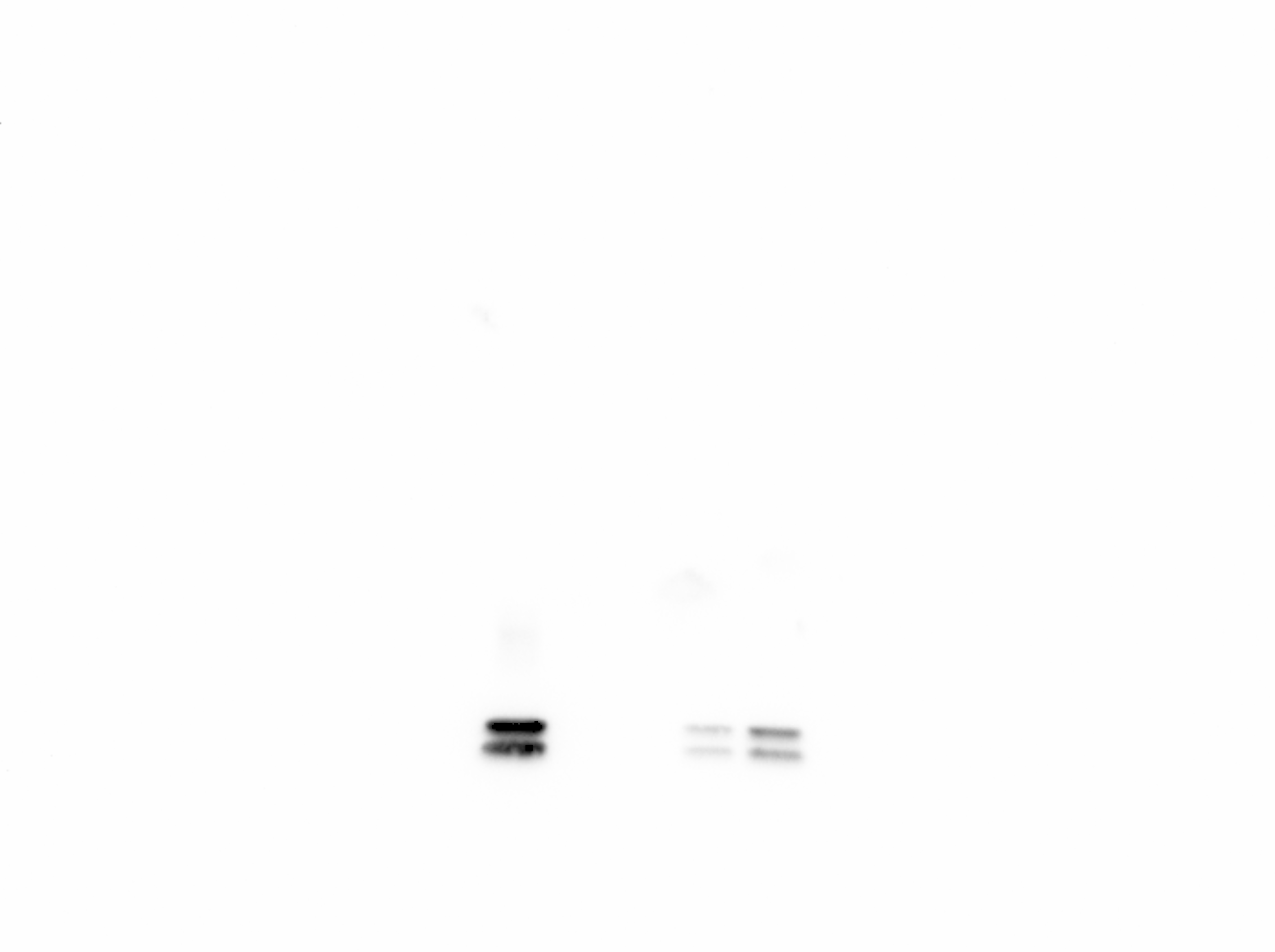

Supplement: Figure 4—figure supplement 1—source data 2. [file elife-80653-fig4-figsupp1-data2.zip › Figure 4-figure supplement 2 -source data 1/ORIGINAL FILES/Panel C H3.tif]

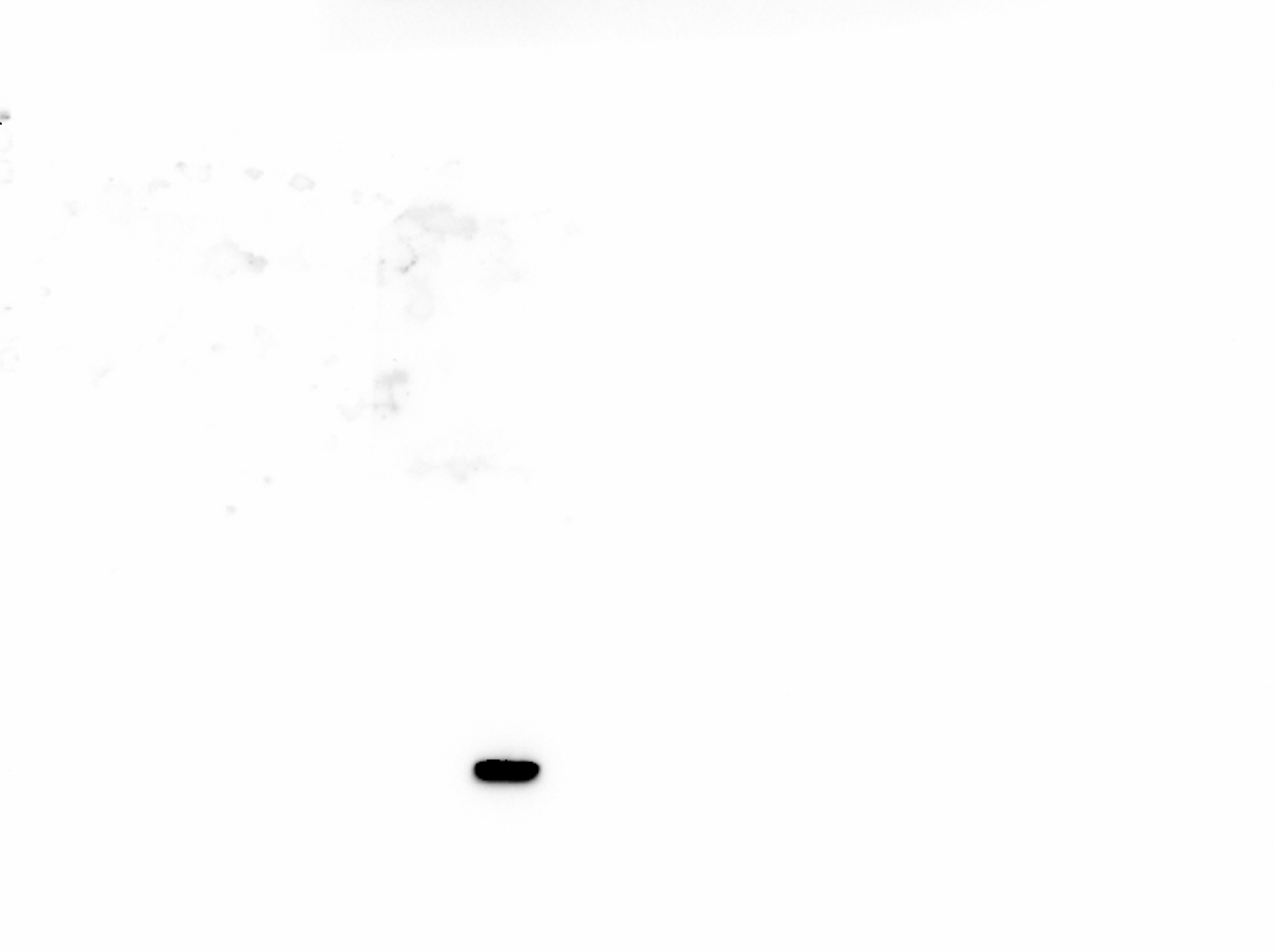

Supplement: Figure 4—figure supplement 1—source data 2. [file elife-80653-fig4-figsupp1-data2.zip › Figure 4-figure supplement 2 -source data 1/ORIGINAL FILES/Panel C H2A.tif]

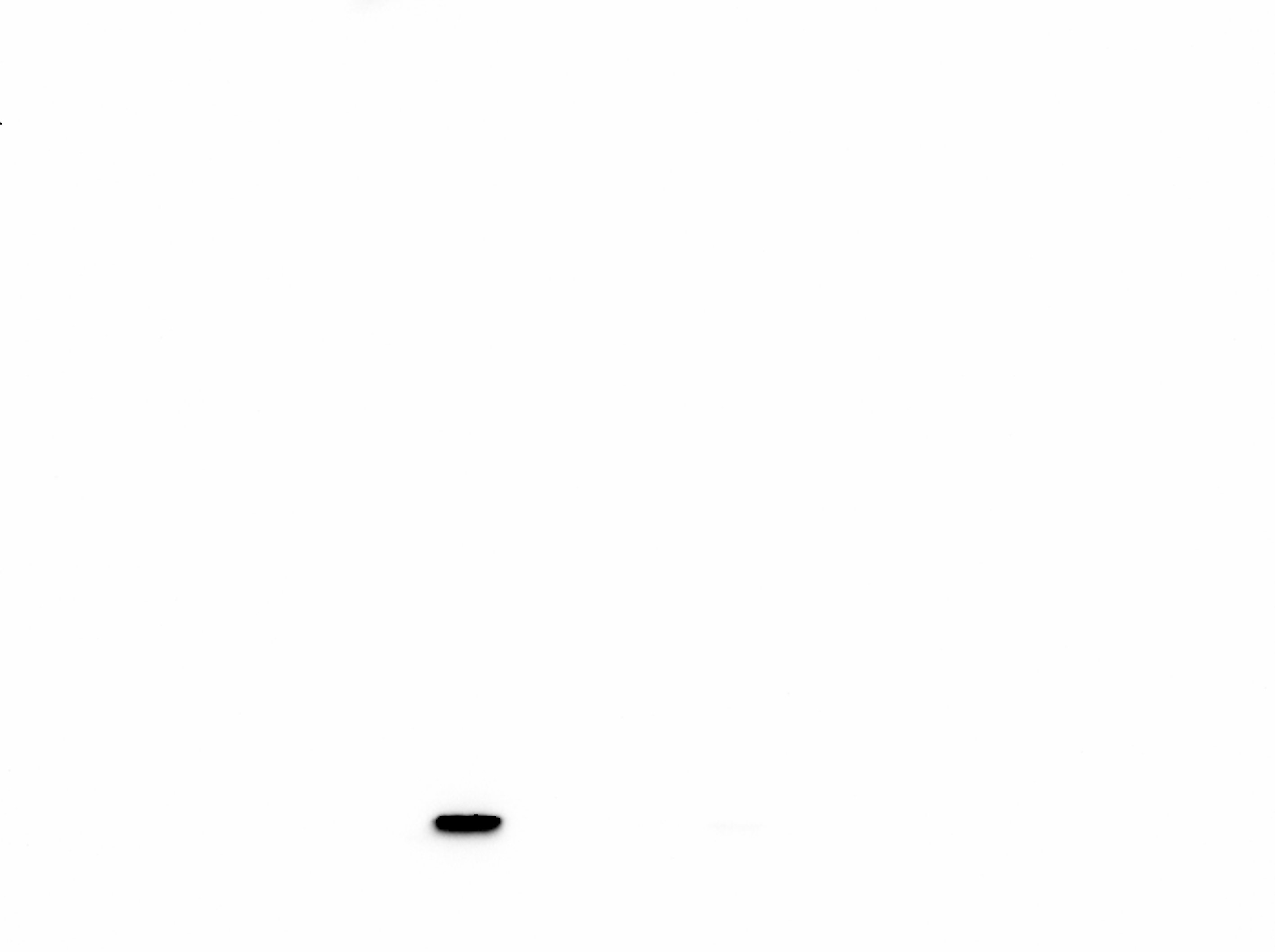

Supplement: Figure 4—figure supplement 1—source data 2. [file elife-80653-fig4-figsupp1-data2.zip › Figure 4-figure supplement 2 -source data 1/ORIGINAL FILES/Panel C H4.tif]

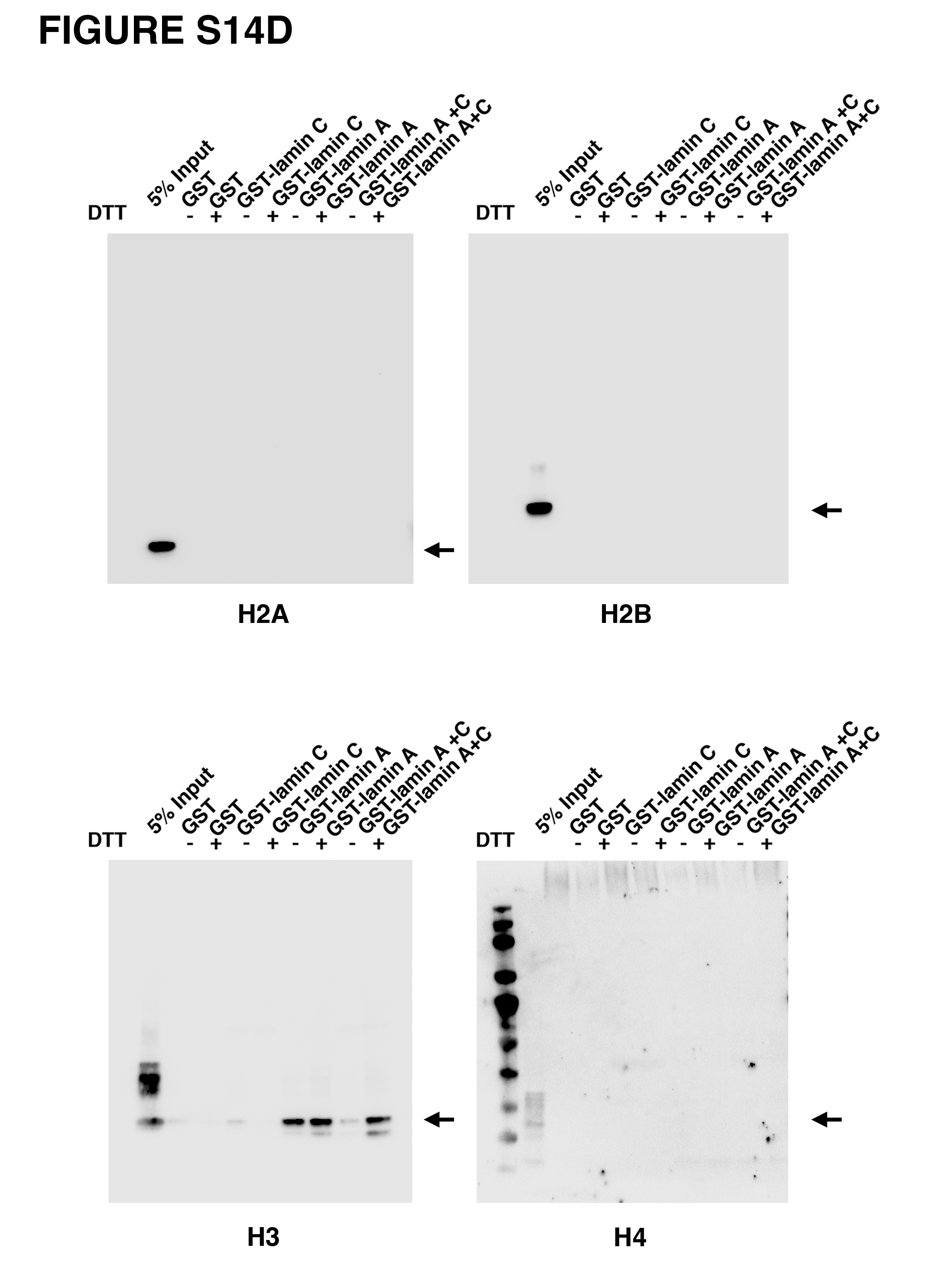

Supplement: Figure 4—figure supplement 1—source data 3. [file elife-80653-fig4-figsupp1-data3.zip › Figure 4-figure supplement 3 -source data 1 /Panel D.tif]

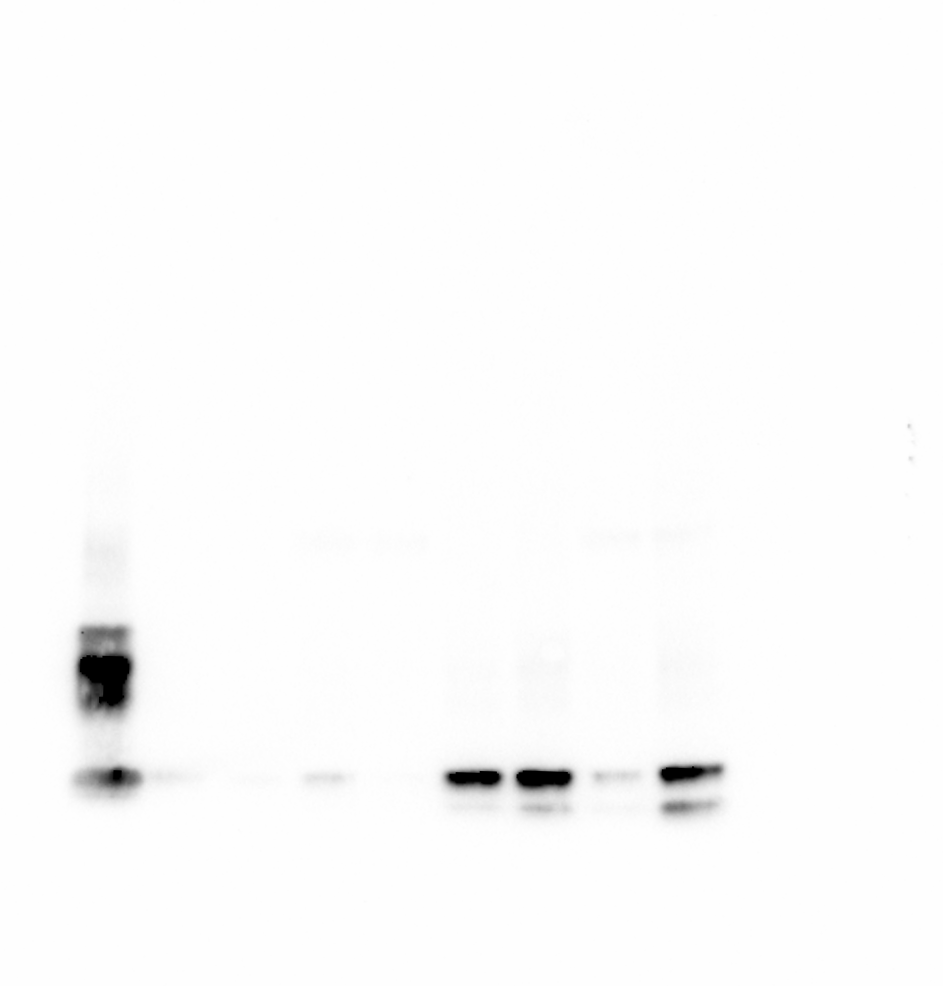

Supplement: Figure 4—figure supplement 1—source data 3. [file elife-80653-fig4-figsupp1-data3.zip › Figure 4-figure supplement 3 -source data 1 /ORIGINAL FILES /Panel D H3.tif]

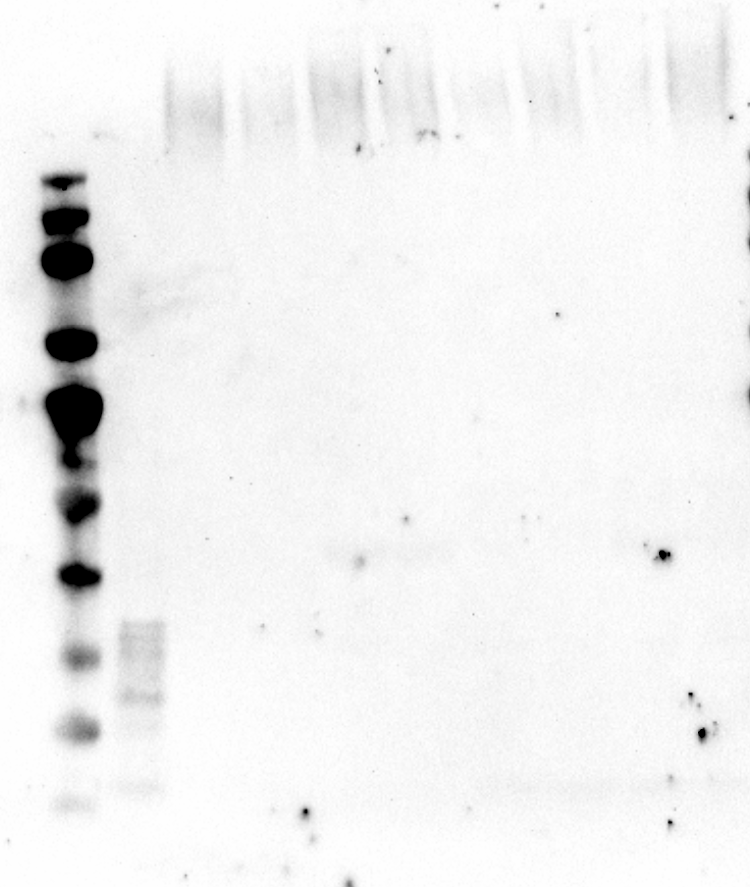

Supplement: Figure 4—figure supplement 1—source data 3. [file elife-80653-fig4-figsupp1-data3.zip › Figure 4-figure supplement 3 -source data 1 /ORIGINAL FILES /Panel D H4.tif]

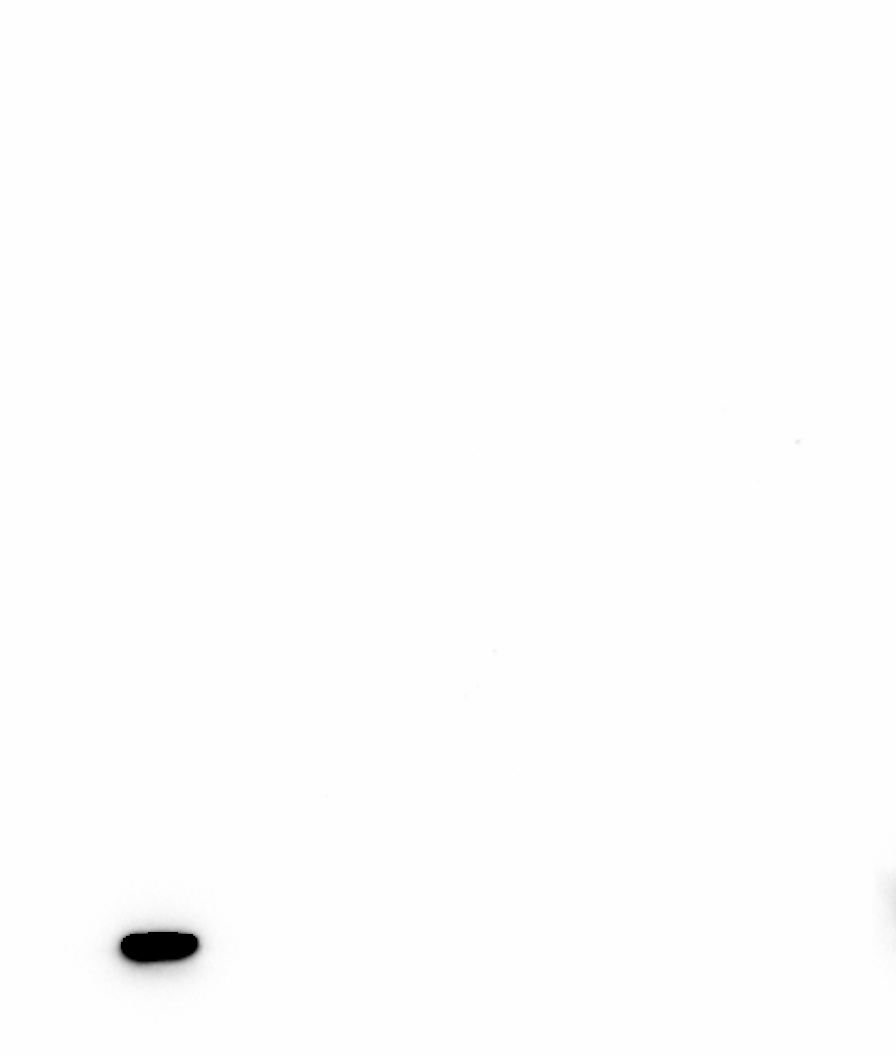

Supplement: Figure 4—figure supplement 1—source data 3. [file elife-80653-fig4-figsupp1-data3.zip › Figure 4-figure supplement 3 -source data 1 /ORIGINAL FILES /Panel D H2A.tif]

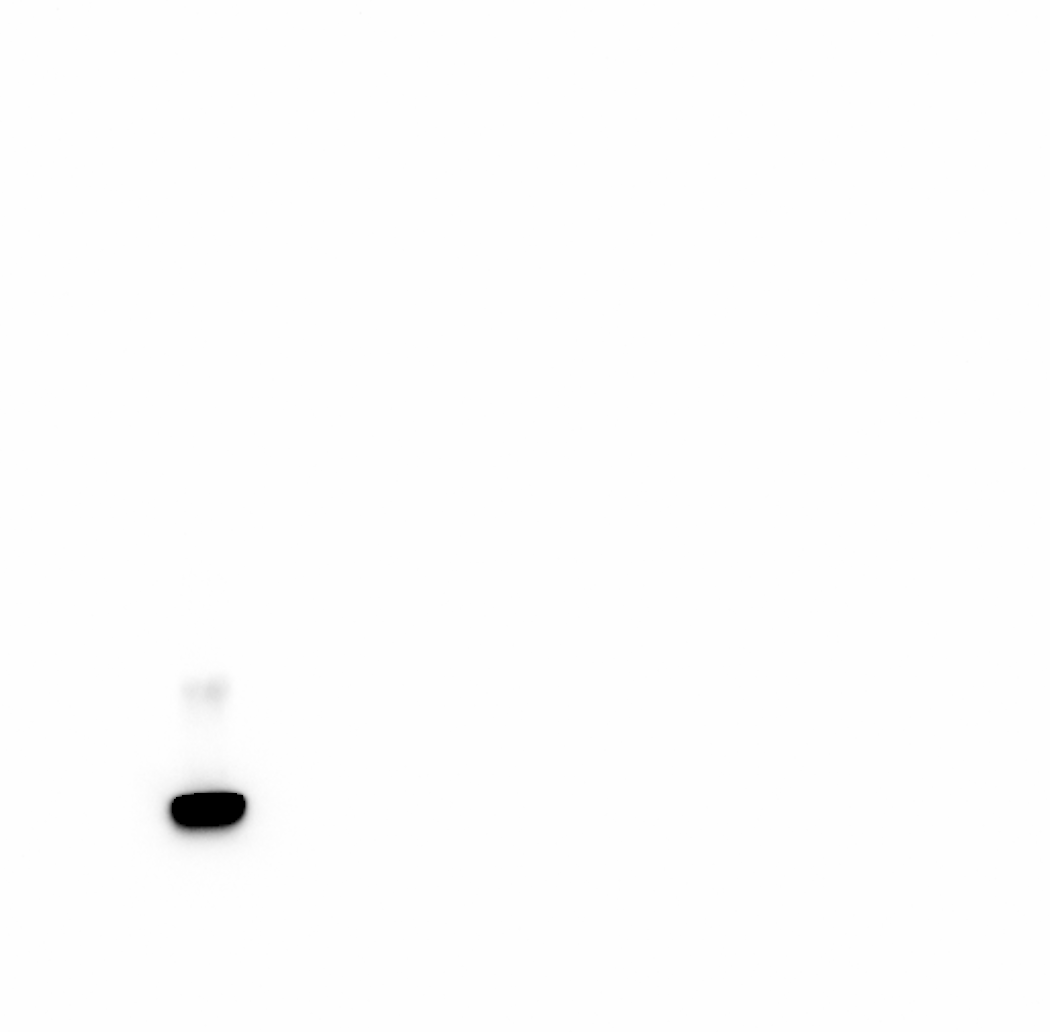

Supplement: Figure 4—figure supplement 1—source data 3. [file elife-80653-fig4-figsupp1-data3.zip › Figure 4-figure supplement 3 -source data 1 /ORIGINAL FILES /Panel D H2B.tif]

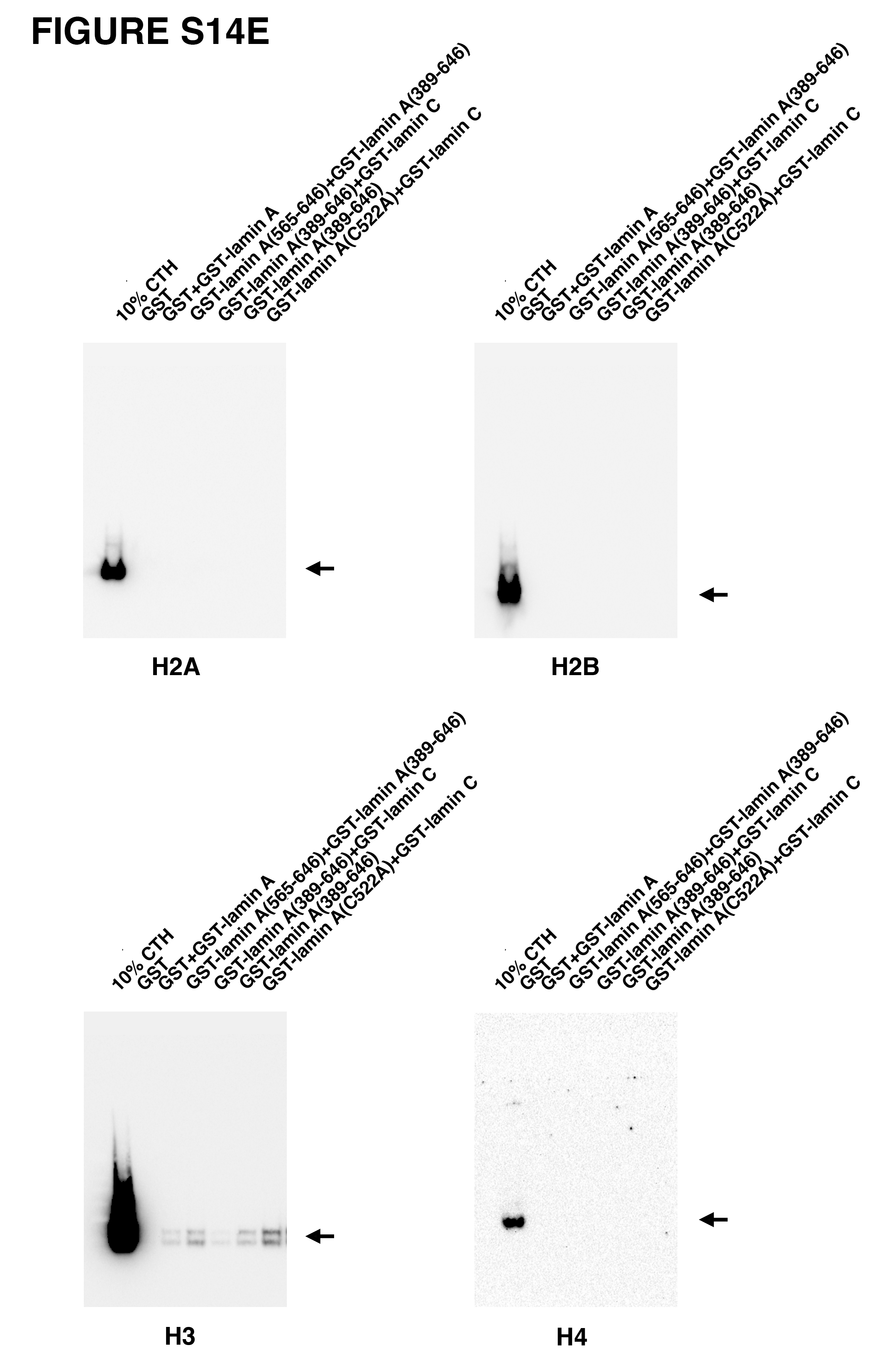

Supplement: Figure 4—figure supplement 1—source data 4. [file elife-80653-fig4-figsupp1-data4.zip › Figure 4-figure supplement 4 -source data 1 /Panel E.tif]

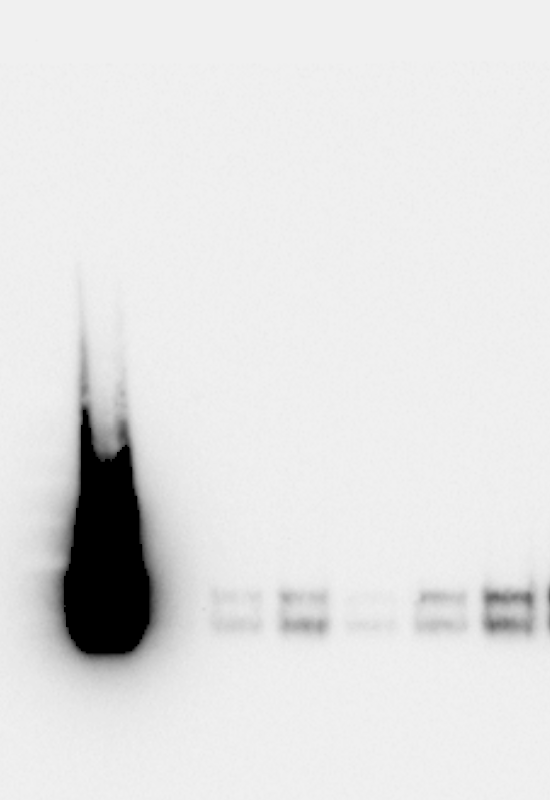

Supplement: Figure 4—figure supplement 1—source data 4. [file elife-80653-fig4-figsupp1-data4.zip › Figure 4-figure supplement 4 -source data 1 /ORIGINAL FILES/Panel E H3.tif]

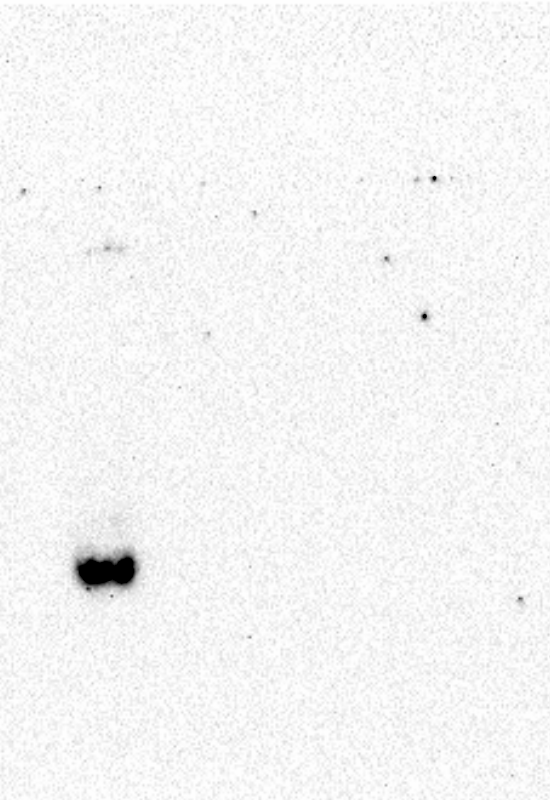

Supplement: Figure 4—figure supplement 1—source data 4. [file elife-80653-fig4-figsupp1-data4.zip › Figure 4-figure supplement 4 -source data 1 /ORIGINAL FILES/Panel E H4.tif]

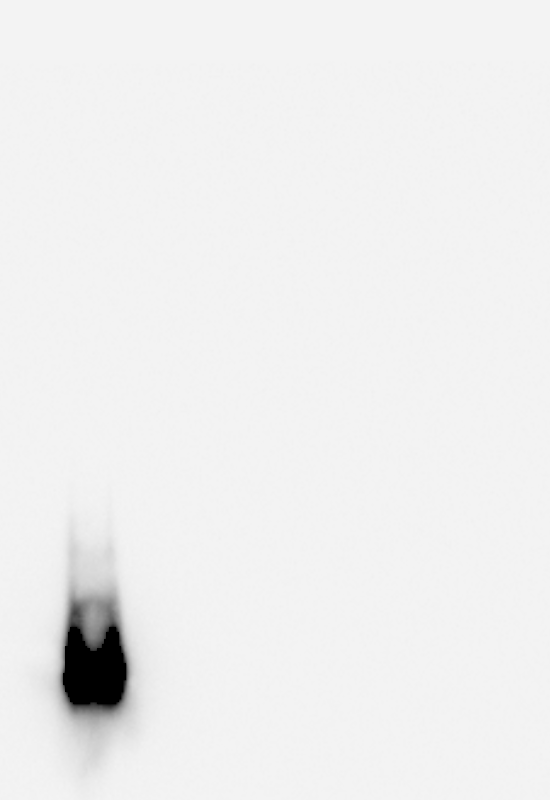

Supplement: Figure 4—figure supplement 1—source data 4. [file elife-80653-fig4-figsupp1-data4.zip › Figure 4-figure supplement 4 -source data 1 /ORIGINAL FILES/Panel E H2b.tif]

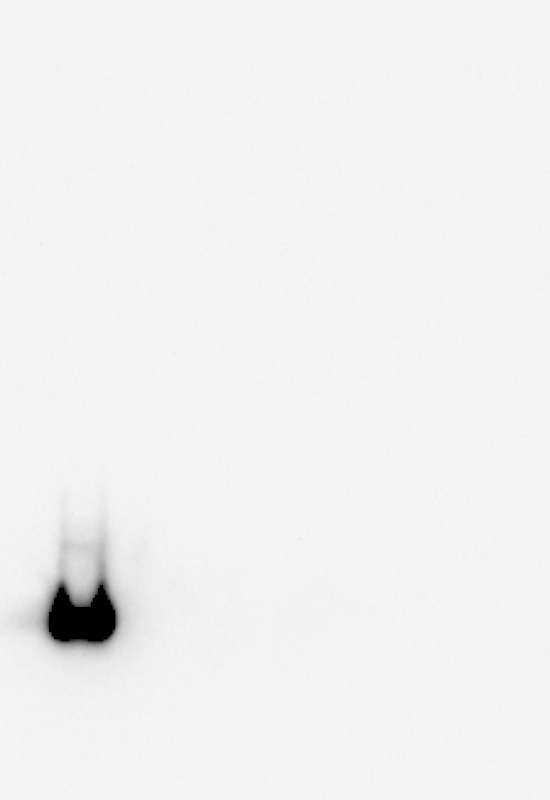

Supplement: Figure 4—figure supplement 1—source data 4. [file elife-80653-fig4-figsupp1-data4.zip › Figure 4-figure supplement 4 -source data 1 /ORIGINAL FILES/Panel E H2A.tif]
